# Transcriptome-guided GLP-1 receptor therapy rescues metabolic and behavioral disruptions in a Bardet-Biedl syndrome mouse model

**DOI:** 10.1172/JCI184636

**Published:** 2025-04-15

**Authors:** Arashdeep Singh, Naila Haq, Mingxin Yang, Shelby Luckey, Samira Mansouri, Martha Campbell-Thompson, Lei Jin, Sofia Christou-Savina, Guillaume de Lartigue

**Affiliations:** 1 Monell Chemical Senses Center, Philadelphia, Pennsylvania, USA.; 2 Department of Neuroscience, University of Pennsylvania, Philadelphia, Pennsylvania, USA.; 3 Department of Pharmacodynamics, College of Pharmacy, University of Florida, USA.; 4 Department of Genetics and Genomic Medicine, University College London, United Kingdom.; 5 Department of Medicine and; 6 Department of Pathology, Immunology and Laboratory Medicine, College of Medicine, University of Florida, USA.

**Keywords:** Endocrinology, Metabolism, Macrophages, Monogenic diseases, Obesity

## Abstract

Bardet-Biedl syndrome (BBS), a ciliopathy characterized by obesity, hyperphagia, and learning deficits, arises from mutations in *Bbs* genes. Exacerbated symptoms occur with mutations in genes encoding the BBSome, a complex regulating primary cilia function. We investigated the mechanisms underlying BBS-induced obesity using a *Bbs5*-knockout (*Bbs5^–/–^*) mouse model. *Bbs5^–/–^* mice were characterized by hyperphagia, learning deficits, glucose/insulin intolerance, and disrupted metabolic hormones, phenocopying human BBS. White adipose tissue in these mice had a unique immunophenotype, with increased proinflammatory macrophages and dysfunctional Tregs, suggesting a mechanism for adiposity distinct from those of typical obesity models. Additionally, *Bbs5^–/–^* mice exhibited pancreatic islet hyperplasia but failed to normalize blood glucose, suggesting defective insulin action. Hypothalamic transcriptomics revealed dysregulation of endocrine signaling pathways, with functional analyses confirming defects in insulin, leptin, and cholecystokinin (CCK) signaling, while glucagon-like peptide-1 receptor (GLP-1R) responsiveness was preserved. Notably, treatment with a GLP-1RA effectively alleviated hyperphagia and body weight gain, improved glucose tolerance, and regulated circulating metabolic hormones in *Bbs5^–/–^* mice. This study suggests that *Bbs5^–/–^* mice represent a valuable translational model of BBS for understanding pathogenesis and developing better treatments. Our findings highlight the therapeutic potential of GLP-1RAs for managing BBS-associated metabolic dysregulation, indicating that further investigation for clinical application is warranted.

## Abstract 2

New mouse model of Bardet-Biedl Syndrome, a rare genetic disease, reveals potential therapeutic targets via transcriptomics and demonstrates proof-of-principle efficacy for GLP1 receptor agonist treatment.

## Graphical abstract

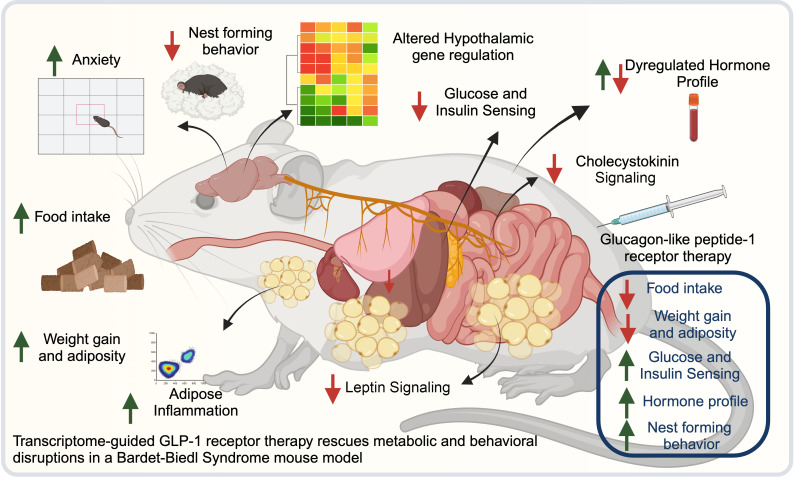

## Introduction

Bardet-Biedl syndrome (BBS) is a rare and debilitating genetic disorder characterized by malfunctions in primary cilia, sensory organelles that play a crucial role in cellular signaling and homeostasis (1). Affecting approximately 1 in 100,000 to 160,000 individuals, BBS presents as a clinically heterogeneous array of symptoms including obesity, learning impairments, retinal dystrophy, and kidney dysfunction (2). The syndrome poses a significant clinical challenge due to its complex genetic etiology, with pathogenic mutations identified in more than 25 genes. These mutations impair the structure and function of cilia, leading to diverse and often severe phenotypic manifestations. Despite substantial advances in genetic and molecular research, effective treatments for BBS remain elusive, largely due to the limited understanding of the underlying pathophysiology.

Central to BBS pathophysiology is the BBSome, a multisubunit protein complex essential for trafficking of receptors within cilia — a function critical for cellular communication and sensing (3). The BBSome comprises 8 core proteins (BBS1, BBS2, BBS4, BBS5, BBS7, BBS8, BBS9, and BBS18) that operate collectively to regulate the transport of receptor molecules within cilia. Disruptions in BBSome assembly or function can impair receptor signaling, leading to the hallmark features of BBS. Among these core components, BBS5 is a compelling target for investigation, as it is highly conserved (4) and plays a critical role in ciliary protein trafficking (2, 5), but it remains an understudied component of the BBSome (6). Mutations in *Bbs5* are estimated to account for 2%–4% of BBS cases (3, 4). Recent studies suggest that *Bbs5* mutations lead to disruptions in retinal health (7) and neuronal morphology (8). However, its broader role in physiological and metabolic function remains poorly characterized.

Given that obesity is a hallmark of BBS and a significant driver of its associated comorbidities, a deepening understanding of BBS5’s role in metabolism could yield critical insights into disease pathogenesis. Our study utilizes *Bbs5*-knockout (*Bbs5^–/–^*) mice as a model to address the knowledge gap by characterizing the physiological impact of BBS5 disruption on cellular and whole-organism metabolism. By elucidating the molecular pathways governed by BBS5, we aim to uncover potential therapeutic targets for treating BBS and related ciliopathies.

## Results

### Bbs5^–/–^ mice recapitulate key clinical metabolic dysregulations of BBS.

**Figure 1:**
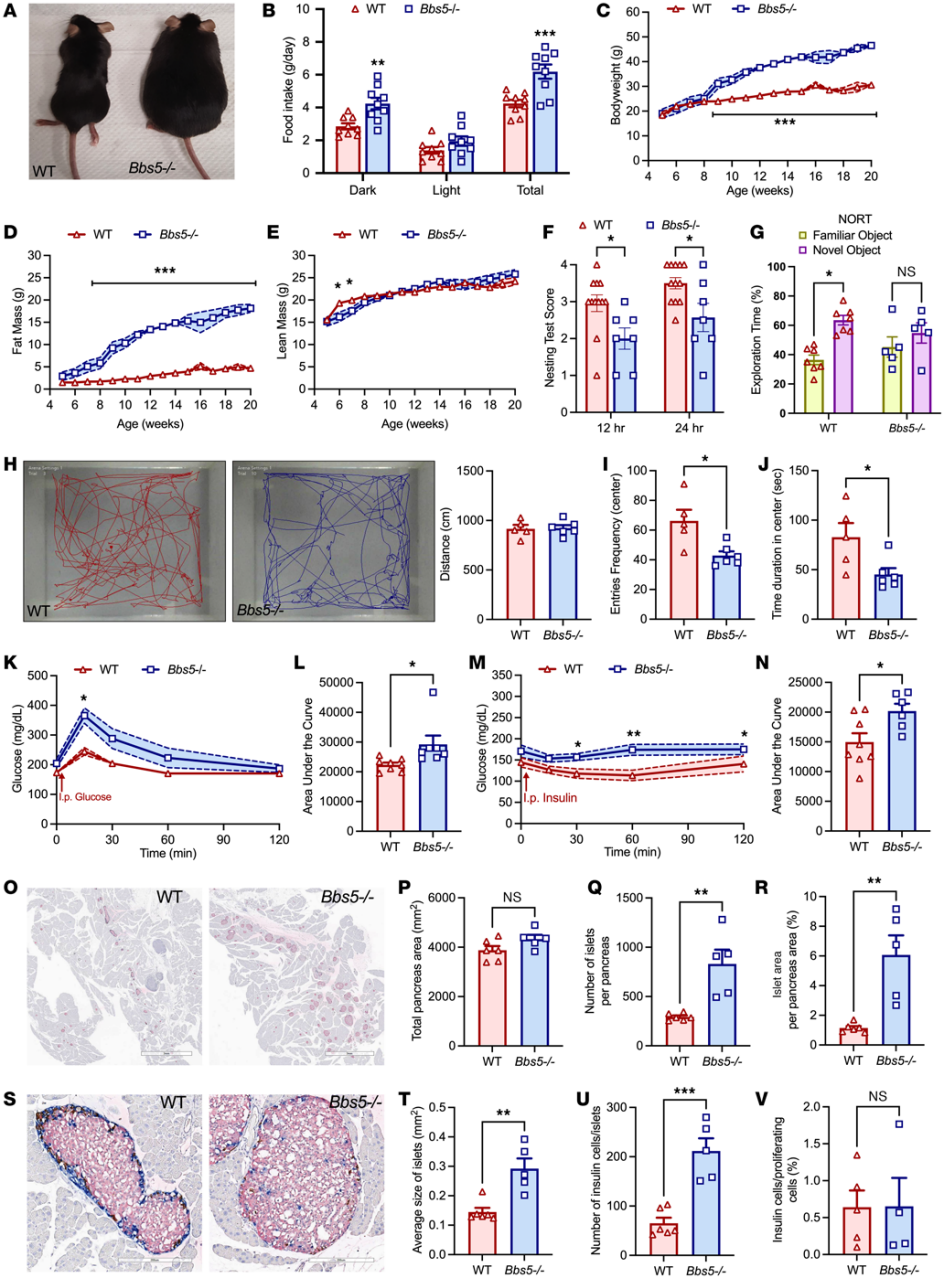
Adult *Bbs5*-null mice are morbidly obese, hyperphagic, glucose intolerant, and have behavioral and learning impairments. (**A**) Representative image of 12-week-old ad libitum chow-fed male WT C57BL/6J and *Bbs5^–/–^* mice. (**B**) Average cumulative daily ad libitum food intake during 12-hour dark and light and 24-hour periods in 10- to 18-week-old mice (*n* = 9 per group). (**C**–**F**) Weekly body weight (**C**), fat mass (**D**), and lean mass (**E**) during development in 5- to 20-week-old male mice (*n* = 5–12 per group). (**F**) Nest-building score after 12 and 24 hours upon provision of cotton-pressed nestlets for 12- to 18-week-old mice (*n* = 7–12 per group). (**G**) Percent exploration time with familiar or novel objects in the NOR paradigm (*n* = 5–8 per group). (**H**) Representative traces of WT and *Bbs5^–/–^* mice during 5-minute open-field test. (**H**–**J**) Distance traveled (**H**), number of entries (**I**), and time duration in the center (**J**) in 14- to 18-week-old mice (*n* = 6 per group). (**K** and **L**) Intraperitoneal glucose tolerance (**K**) and area under the curve (**L**) in 11- to 18-week-old mice (*n* = 6–8 per group). (**M** and **N**) Insulin tolerance (**M**) and area under the curve (**N**) in 11- to 18-week-old mice (*n* = 6–8 per group). (**O**–**V**) Representative images of islet immunohistochemistry for insulin, glucagon, and somatostatin staining from pancreas sections (**O**; scale bars: 3 mm); quantitative analyses of total pancreas area (**P**); number of islets (**Q**); percent islet area (**R**); higher-magnification images showing normal distribution of central β cells (pink), with peripherally located glucagon (blue) and somatostatin (brown) cells (**S**; scale bars: 200 μm); average islet size (**T**); number of insulin cells per islet (**U**); and percent insulin cells relative to proliferating cells (**V**) of 16- to 18-week-old mice (*n* = 5–6 per group). Data in **B**–**G**, **K**, and **M** were analyzed using repeated-measure 2-way ANOVA with Benjamini, Krieger, and Yekutieli post hoc test (FDR = 0.05) to compare individual time points. Data in **H**–**J**, **L**, **N**, **P**–**R**, and **T**–**V** were analyzed using Student’s 2-sided, 2-tailed *t* test. Data are mean ± SEM from chow-fed WT and *Bbs5^–/–^* male mice; *P < 0.05; **P < 0.01; ***P < 0.001.

Clinically, BBS presents with a high prevalence of early-onset obesity (70%–90%) linked to hyperphagia (9). To investigate the specific role of BBS5 in metabolic disease, we used a *Bbs5^–/–^* mouse (7) maintained on a homogeneous C57BL/6J background born from heterozygous parents. Although *Bbs5^–/–^* mice showed no differences in body weight at weaning compared to age-matched WT or heterozygous littermate controls, the KO mice began gaining adiposity at a significantly faster rate after 4 weeks of age, eventually becoming visibly larger than mice in both control groups (Figure 1A) *Bbs5^–/–^* mice exhibited significant hyperphagia in both the light and dark phases compared with WT controls (Figure 1B), leading to increased body weight from 8 weeks of age (Figure 1C) and fat mass compared with WT littermate controls starting from 6 weeks of age (Figure 1D). Notably lean mass remained comparable in the 2 groups (Figure 1E).

In a clinical study, neuropsychological deficiencies, including reduced intelligence quotient, impaired fine-motor function, social skill deficits, and decreased olfaction, were observed in patients with BBS (10). In this study, *Bbs5^–/–^* mice showed impaired nest-building behavior (Figure 1F), indicative of potential learning and/or motor deficits. In a novel object recognition (NOR) paradigm, *Bbs5^–/–^* mice spent less time exploring the novel object than did WT littermates, which was indicative of memory retention deficits (Figure 1G). In an open-field test, there were no differences in locomotion (Figure 1H), but *Bbs5^–/–^* mice exhibited reduced center entries (Figure 1I) and time spent in the center (Figure 1J) compared with WT littermates. We observed no additional behavioral disparities in elevated plus maze (EPM) (Supplemental Figure 1, A and B; supplemental material available online with this article; https://doi.org/10.1172/JCI184636DS1) or Y-maze experiments (Supplemental Figure 1C), suggesting that the impaired entries into the center were caused by deficits in exploratory behavior rather than heightened anxiety. We observed during feeding experiments that *Bbs5^–/–^* mice failed to learn to eat from feeders, further supporting the idea of potential learning deficits. Based on our previous findings, we postulate that the behavioral and learning impairments observed in patients with BBS could be due to synaptic dysfunction in principal hippocampal neurons (8); however, there is still a need for direct electrophysiological studies to assess synaptic function in *Bbs5^–/–^* mice.

**Figure 2:**
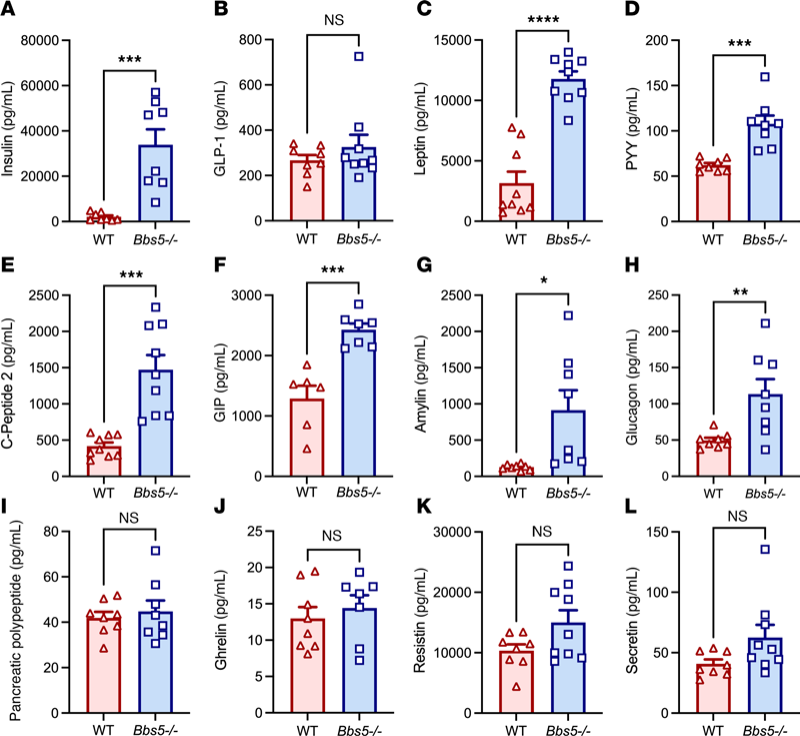
Adult *Bbs5*-null mice have dysregulated plasma levels of circulating metabolic hormones. (**A**–**L**) Plasma concentrations of insulin (**A**), GLP-1 (active) (**B**), leptin (**C**), PYY (**D**), C-peptide 2 (**E**), GIP (total) (**F**), amylin (active) (**G**), glucagon (**H**), PP (**I**), ghrelin (**J**), resistin (**K**), and secretin (**L**) in 15- to 22-week-old ad libitum chow-fed male and female WT C57BL/6J and *Bbs5^–/–^* mice (*n* = 9 per strain). Data in **A**–**L** were analyzed using Student’s 2-sided, 2-tailed *t* test. Data are mean ± SEM from WT and *Bbs5^–/–^* mice; *P < 0.05; **P < 0.01; ***P < 0.001; ****P < 0.0001.

Consistent with the link to diabetes progression in ciliopathy (11), and the higher diabetes prevalence in patients with BBS patients compared with control individuals with obesity (12), *Bbs5^–/–^* mice exhibited impaired glucose clearance. This was evidenced by elevated circulating glucose levels following glucose administration (Figure 1K) and a higher area under the curve (Figure 1L) compared with those in WT controls. Similarly, insulin injections failed to reduce circulating glucose levels in *Bbs5^–/–^* mice (Figure 1M), resulting in a higher area under the curve (Figure 1N). The presence of primary cilia on islet cells is conserved across species (13) and has been shown to be critical for signaling of specific GPCRs to regulate islet insulin and glucagon secretion (14). To elucidate the mechanisms underlying glucose and insulin intolerance, we conducted a morphological assessment of pancreatic tissue and islet cell architecture in *Bbs5^–/–^* and WT controls (Figure 1, O–V). *Bbs5^–/–^* mice had a pancreas size similar to that in WT mice (Figure 1P), but displayed pancreatic islet hyperplasia (Figure 1Q), with a greater percentage of islet relative to pancreas area (Figure 1R), larger average islet size (Figure 1, S and T), and an increased number of insulin cells per islet (Figure 1U). We observed no significant changes in the percentage of insulin cells relative to proliferating cells between groups (Figure 1V). Consistent with these changes in islet morphology, *Bbs5^–/–^* mice had elevated circulating insulin levels (Figure 2A). Thus, like in other ciliopathy models (7, 15, 16), ablation of *Bbs5* led to aberrant pancreatic morphology, defective insulin signaling, and impaired glucose homeostasis. These data are consistent with evidence that primary cilia present in insulin-producing β cells are implicated in regulating glucose metabolism, insulin signaling, and insulin secretion (14, 16, 17).

Importantly, the metabolic and behavioral abnormalities observed in male BBS5*^–/–^* mice were recapitulated in females (Supplemental Figure 1, D–J), suggesting that there were no sex differences in disease manifestation. Additionally, neither male (Supplemental Figure 1, K–P) nor female (Supplemental Figure 1, Q–V) heterozygous *Bbs5^+/–^* mice displayed any discernable phenotypic changes compared with WT controls. Thus, key metabolic and neurobehavioral dysregulations of BBS were effectively recapitulated in *Bbs5^–/–^* mice, which offer a robust platform for dissecting BBS pathophysiology and developing potential therapeutic strategies.

To better understand the mechanisms underlying the metabolic disruptions in BBS, we investigated changes in a range of metabolic hormones in ad libitum fed WT and *Bbs5^–/–^* mice. Neither male nor female *Bbs5^–/–^* mice exhibited significant differences in circulating levels of glucagon-like peptide–1 (GLP-1); however, they had elevated circulating levels of insulin, leptin, peptide YY (PYY), C-peptide 2, gastric inhibitory polypeptide (or glucose-dependent insulinotropic polypeptide [GIP]), amylin, and glucagon compared with WT controls (Figure 2, A–H). Additionally, no significant differences were observed in circulating pancreatic polypeptide (PP), ghrelin, resistin, and secretin levels (Figure 2, I–L). These findings provide a comprehensive endocrine profile for *Bbs5^–/–^* mice, revealing specific hormonal alterations that may contribute to the metabolic phenotype associated with BBS.

### White adipose tissue of Bbs5^–/–^ mice exhibits an increased proinflammatory immunophenotype.

**Figure 3:**
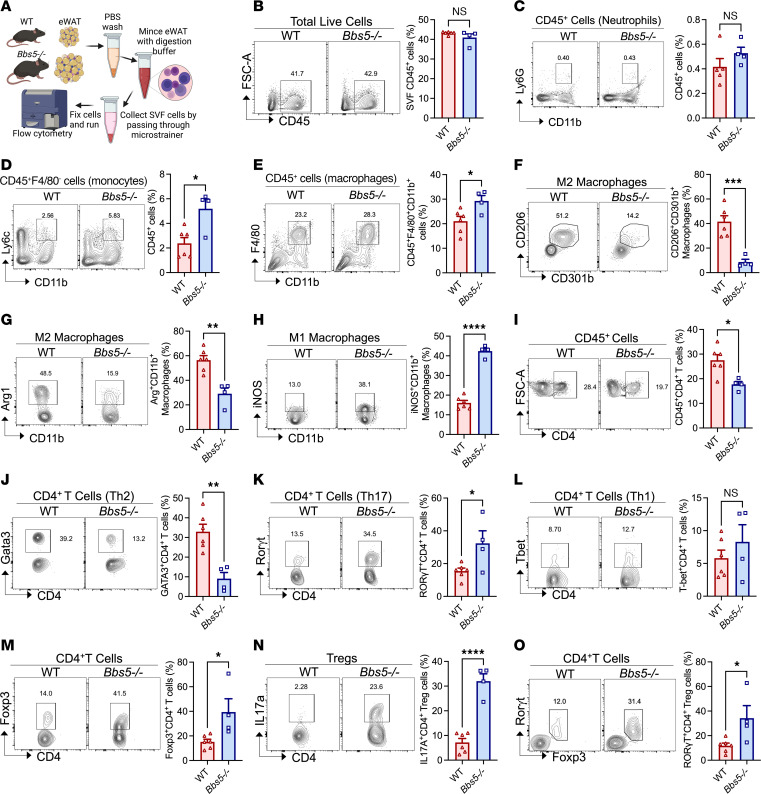
Adult *Bbs5-*null mice have a proinflammatory white adipose immunophenotype. (**A**) Schematic workflow diagram of the isolation of immune cells in the stromal vascular fraction (SVF) of eWAT from male WT and *Bbs5^–/–^* mice by flow cytometry. (**B**–**O**) Flow cytometry analysis of CD45^+^ cells (**B**), neutrophils (**C**), Ly6G^hi^ monocytes (**D**), total macrophages (**E**), CD206^+^CD301b^+^ M2 macrophages (**F**), Arg^+^ M2 macrophages (**G**), iNOS^+^ M1 macrophages (**H**), CD45^+^CD4^+^ T cells (**I**), Gata3 Th2 cells (**J**), Rorγt Th17 cells (**K**), T-bet Th1 cells (**L**), Foxp3^+^ Tregs (**M**), IL-17^+^ Tregs (**N**), and Rorγt^+^Foxp3^+^ Tregs (**O**) in eWAT of 18- to 22-week-old male WT and *Bbs5^–/–^* mice (*n* = 4–6 per group). Data are representative of 2 independent experiments. Data in **B**–**O** were analyzed using Student’s 2-sided, 2-tailed *t* test. Data are mean ± SEM from male WT and *Bbs5^–/–^* mice; *P < 0.05; **P < 0.01; ***P < 0.001; ****P < 0.0001.

Primary cilia are highly dynamic organelles that play a vital role in immune (18, 19) and adipocyte function (20, 21). Given the higher fat mass of *Bbs5^–/–^* mice, we investigated how BBS5 affects adipose tissue immunity in these mice. Evaluation of epididymal white adipose tissue (eWAT) immune cell populations (Figure 3A) revealed no significant differences in *Bbs5^–/–^* compared with WT total leukocytes and neutrophils (Figure 3, B and C). However, *Bbs5^–/–^* mice exhibited a striking increase in eWAT monocytes (Figure 3D). Macrophages, crucial regulators of inflammation (22), were also elevated (Figure 3E). Notably, M2 (antiinflammatory) macrophage levels were reduced (Figure 3, F and G), and M1 (proinflammatory) macrophage levels were significantly higher (Figure 3H). This shift was recapitulated in female *Bbs5^–/–^* mice (Supplemental Figure 2, B–D). These findings suggest that *Bbs5* mutations disrupt adipose tissue homeostasis by altering immune cells.

Recent studies have implicated Tregs, a subset of CD4^+^ T cells, in promoting antiinflammatory responses within eWAT (23). Consistent with this, *Bbs5^–/–^* mice displayed a decrease in total eWAT CD4^+^ T cells compared with WT controls (Figure 3I). Further analysis revealed a decrease in Gata3^+^ Th2 cells (Figure 3J), while Rorγt^+^ Th17 cells were conversely elevated (Figure 3K), but T-bet^+^ Th1 cell levels did not differ from those of WT controls (Figure 3L). Notably, Treg numbers were paradoxically higher in *Bbs5^–/–^* mice than in WT controls (Figure 3M). However, these Tregs exhibited increased expression of IL-17a and Rorγt (Figure 3, N and O), markers associated with Th17 cells rather than the immunosuppressive Tregs This dysfunctional Treg phenotype aligns with the chronic, low-grade inflammatory state observed in the eWAT of *Bbs5^–/–^* mice. Female *Bbs5^–/–^* mice displayed a similar trend in Treg profiles (Supplemental Figure 2, E and F).

### Bbs5 mutation significantly alters hypothalamic transcriptomics and predicts endocrine dysfunction.

**Figure 4:**
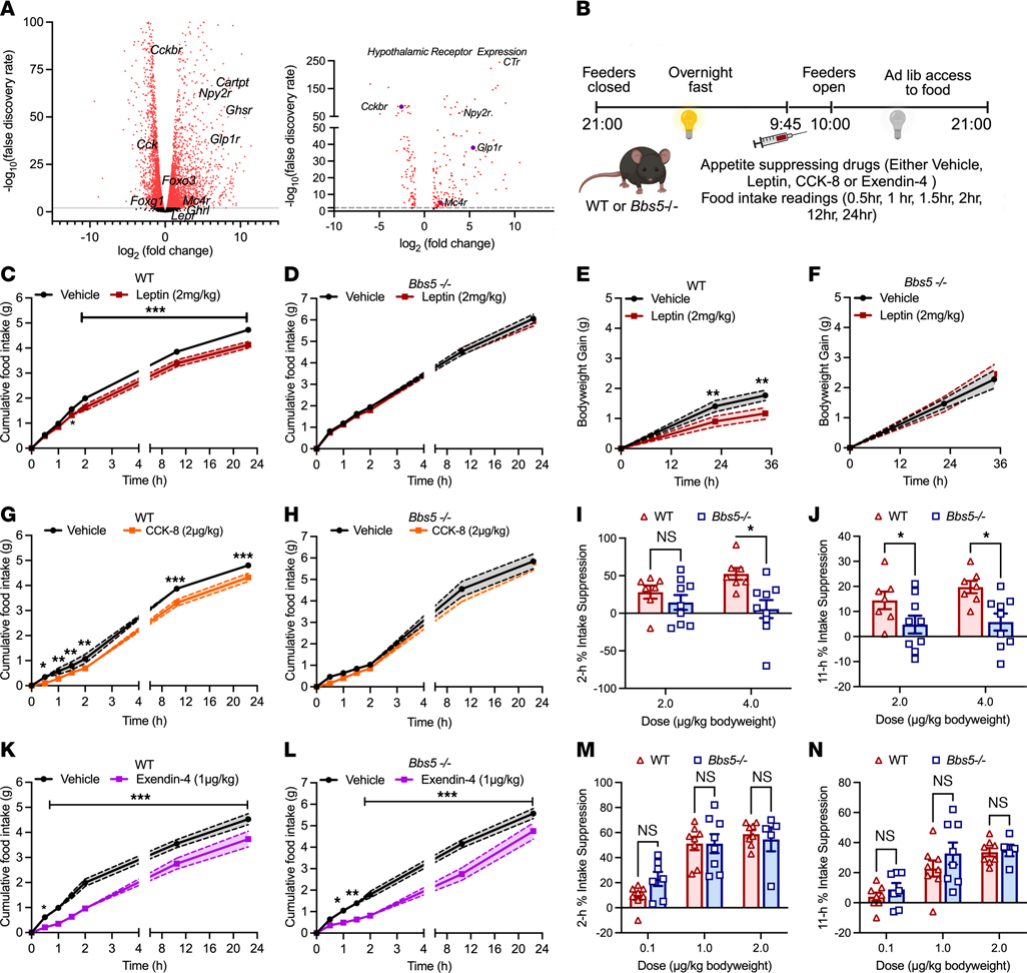
Hypothalamic RNA transcriptomics predicts leptin and CCK resistance but retention of GLP-1 response in male *Bbs5*-null mice. (**A**) Volcano plot of differential expression changes, plotting the log fold change (FC) in receptors in the hypothalamus in 12-week-old male *Bbs5^–/–^* relative to WT mice (*n* = 2–3 per group). Red dots represent significant differences; black, nonsignificant. (**B**) Schematic experimental design to determine anorexigenic effects of leptin, CCK-8, and GLP-1RA exendin-4 in overnight-fasted male WT and *Bbs5^–/–^* mice. (**C** and **D**) The effect of i.p. leptin (red, 2 mg/kg body weight) or vehicle (black) injection on cumulative ad libitum food intake in 12- to 18-week-old male WT (**C**) and *Bbs5^–/–^* (**D**) mice over 24 hours (*n* = 9 per group). (**E** and **F**) Body weight gain in WT (**E**) and *Bbs5^–/–^* (**F**) mice at 24 and 36 hours after leptin injections. (**G** and **H**) Effect of i.p. CCK-8 (orange, 2 μg/kg body weight) or vehicle (black) injection on cumulative ad libitum food intake in 12- to 18-week-old male WT (**G**) and *Bbs5^–/–^* (**H**) mice over 24 hours (*n* = 7–9 per group). (**I** and **J**) Food intake suppression after 2 (**I**) or 11 hours (**J**) in 12- to 18-week-old male WT and *Bbs5^–/–^* mice following CCK-8 (2 or 4 μg/kg body weight) relative to saline injection (*n* = 7–9 per group). (**K** and **L**) Effect of i.p. exendin-4 (purple, 1 μg/kg body weight) or vehicle (black) injection on cumulative ad libitum food intake in 12- to 18-week-old male WT (**K**) and *Bbs5^–/–^* (**L**) mice over 24 hours (*n* = 8 per group). (**M** and **N**) Food intake suppression after 2 (**M**) or 11 hours (**N**) in 12- to 18-week-old male WT and *Bbs5^–/–^* mice following exendin-4 (0.1 or 1 or 2 μg/kg body weight) relative to saline injection (*n* = 8 per group). Data in **C**–**N** were analyzed using repeated-measure 2-way ANOVA with Benjamini, Krieger, and Yekutieli post hoc test (FDR = 0.05) to compare individual time points. Data are mean ± SEM from chow-fed WT (vehicle or drug treated) and *Bbs5^–/–^* (vehicle or drug treated) male mice; nd, no discovery; *P < 0.05; **P < 0.01; ***P < 0.001.

To elucidate the molecular underpinnings of BBS-associated hyperphagia and weight gain, we performed bulk RNA-Seq on the hypothalamus of 5-week-old (pre-obesity) and 12-week-old (post-obesity) *Bbs5^–/–^* mice. Differential gene expression analysis revealed significant alterations in genes associated with primary cilia function at both the pre- and post-obesity stages in *Bbs5^–/–^* mice (Figure 4A, Supplemental Figure 4, A and B, and Supplemental Figure 5A). In pre-obese *Bbs5^–/–^* mice, we found 401 genes to be upregulated and 549 genes downregulated compared with WT controls (Supplemental Figure 4B). Enriched upregulated gene sets for pre-obese mice were associated with protein sorting and translation, cellular disassembly, RNA transport, DNA and RNA metabolism, and GPCR signaling; and the downregulated gene sets included those involved in extracellular structure and hormone metabolic process (Supplemental Figure 4C). In obese *Bbs5^–/–^* mice, differential expression analysis identified 1,539 genes upregulated and 1,182 genes downregulated compared with WT controls (Figure 4A). Upregulated gene sets included those associated with GPCR signaling, neuropeptide receptor interactions and ligand binding, cilia formation, metabolic processes, and endocrine function Supplemental Figure 5B). In contrast, downregulated gene sets included those related to synaptic regulation, channel activity, mitochondrial function, and enzyme activity (Supplemental Figure 5B).

We observed dysregulation of multiple transcription factors in the hypothalamus that are critical for various metabolic functions (Figure 4A), including impaired leptin signaling, a hallmark of BBS (24), and disrupted cholecystokinin (CCK) signaling. Based on these transcriptomic data, we investigated the functional responsiveness of *Bbs5^–/–^* mice to these satiety hormones (Figure 4B). As expected from previous studies (25), leptin administration significantly reduced food intake and body weight in fasted WT controls (Figure 4, C and E). However, *Bbs5^–/–^* mice displayed complete resistance to leptin-induced satiety. This was evidenced by the lack of change in food intake or body weight following leptin injection (Figure 4, D and F). This finding aligns with leptin resistance reported in other BBS models (24, 26) and suggests impaired leptin signaling within the hypothalamus of *Bbs5^–/–^* mice. Importantly, the leptin resistance phenotype was recapitulated in female *Bbs5^–/–^* mice (Supplemental Figure 3, A–D). Interestingly, even heterozygous *Bbs5^+/–^* mice, which displayed normal body composition and energy metabolism compared with WT controls (Supplemental Figure 1, K–V), were resistant to leptin (Supplemental Figure 3, K, L, O, and P), suggesting that leptin resistance may serve as a potential biomarker even in individuals with a single *Bbs* gene mutation.

To further validate the RNA-Seq findings, we examined the functional responsiveness of *Bbs5^–/–^* mice to CCK. Consistent with downregulation of the CCK signaling pathway, exogenous CCK administration (2 or 4 μg/kg bodyweight) effectively reduced food intake in fasted WT controls (Figure 4G), demonstrating functional CCK signaling. However, *Bbs5^–/–^* mice displayed complete resistance to CCK-induced satiety at both doses (Figure 4, H–J). This resistance was also observed in female *Bbs5^–/–^* mice compared with WT controls (Supplemental Figure S3). Notably, heterozygous *Bbs5^+/–^* mice responded normally to CCK, indicating intact CCK signaling in this group (Supplemental Figure 3, M and Q). Thus, as predicted by RNA-Seq, leptin and CCK signaling was compromised in *Bbs5^–/–^* mice, which may explain at least in part the hyperphagic phenotype in BBS.

The RNA-Seq analysis revealed an unexpected upregulation of numerous hypothalamic satiety hormone receptors, suggesting possible therapeutic targets (Figure 4A). Notably, we observed a 2- to 3-fold increase in melanocortin-4 receptor (*Mc4r*) and neuropeptide Y2 receptor (*Npy2r*) expression in *Bbs5^–/–^* mice. Interestingly, we found an 8-fold upregulation of GLP-1 receptor (*Glp1r*). Consistent with the transcriptomic prediction, the GLP-1R agonist (GLP-1RA) exendin-4 (0.1,1, and 2 μg/kg body weight) reduced food intake in both WT and *Bbs5^–/–^* fasted mice (Figure 4, K and L) in a dose-dependent manner (Figure 4, M and N). Similar results were observed in female *Bbs5^–/–^* mice (Supplemental Figure 3, I and J) as well as heterozygous *Bbs5^+/–^* mice (Supplemental Figure 3, N and R). These findings suggest that GLP-1R signaling remains functional in *Bbs5^–/–^* mice and could represent a promising therapeutic target for the hyperphagia and obesity associated with BBS.

### GLP-1RAs improve key clinical metabolic dysregulations in Bbs5^–/–^ mice.

GLP-1RAs are a class of drugs that have demonstrated efficacy in managing obesity and metabolic disorders. GLP-1RAs, including semaglutide, enhance insulin sensitivity, promote satiety, and reduce body weight (27). Our findings demonstrated elevated hypothalamic GLP-1R expression (Figure 4A) and preserved responsiveness to the acute GLP-1RA exendin-4 in *Bbs5^–/–^* mice (Figure 4, L–N), making them a compelling candidate for addressing BBS-associated metabolic dysfunction.

**Figure 5:**
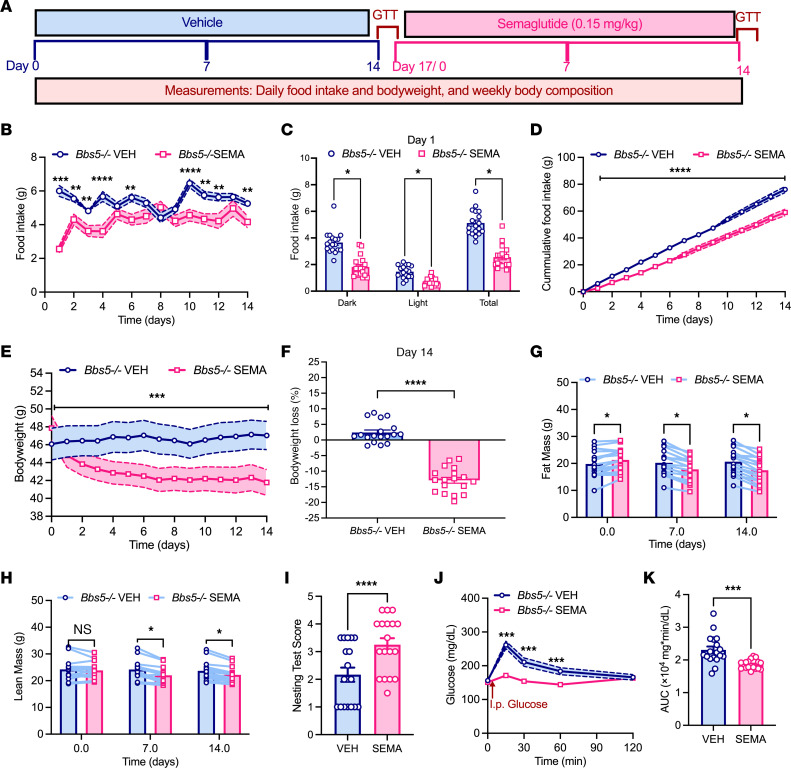
GLP-1RA semaglutide promotes hypophagia-induced weight loss and improves nesting behavior and glucose tolerance in adult *Bbs5*-null mice. (**A**) Schematic diagram of study timeline: obese ad libitum chow-fed *Bbs5^–/–^* mice received daily subcutaneous injections of vehicle (blue) for 14 days, followed by semaglutide (pink, 0.15 mg/body weight) for 14 days; and were observed for changes in feeding, body composition, nest-building behavior, and glucose tolerance. The data in **B**–**K** are from 19- to 30-week-old *Bbs5^–/–^* mice (*n* = 18; 6 males and 12 females). (**B**–**D**) Changes in ad libitum chow intake daily over 24 hours (**B**); average cumulative intake during 12-hour dark and 12-hour light and 24-hour periods (**C**); and average cumulative daily food intake during 14 days of vehicle (VEH) or semaglutide (SEMA) therapy (**D**). (**E**–**H**) Average daily changes in body weight (**E**); average percent weight loss (**F**); and weekly changes in fat mass (**G**) and lean mass (**H**) after semaglutide therapy. (**I**) Nesting score after 12 and 24 hours upon provision of pressed cotton nestlet before and after semaglutide therapy. (**J** and **K**) Intraperitoneal glucose tolerance (**J**) and area under the curve (**K**) before and after semaglutide therapy. Data in **B**–**K** were analyzed using Student’s 2-sided, 2-tailed *t* test or repeated-measure 2-way ANOVA with Benjamini, Krieger, and Yekutieli post hoc test (FDR = 0.05) to compare individual time points. Data are mean ± SEM from vehicle- or drug-treated mice; *P < 0.05; **P < 0.01.

We employed a crossover design in which mixed-sex *Bbs5^–/–^* mice received daily subcutaneous injections of vehicle for 14 days, followed by semaglutide (0.15 mg/kg) for another 14 days (Figure 5A). This dose was chosen based on its reported efficacy in preclinical models of obesity (28). Semaglutide treatment significantly decreased daily food intake in *Bbs5^–/–^* mice, with reductions observed during both the dark and light phases (Figure 5, B and C). This translated to a substantial decrease in cumulative food intake over 14 days (Figure 5D). Consequently, *Bbs5^–/–^* mice treated with semaglutide exhibited rapid weight loss (Figure 5E) and maintained a significant reduction in body weight, exceeding 10% compared with pretreatment levels (Figure 5F). Conversely, during the vehicle treatment arm, *Bbs5^–/–^* mice trended to gain a small amount of weight (Figure 5F). Semaglutide-induced body weight reduction was accompanied by a decrease in fat mass (Figure 5G), with a smaller decrease in lean mass (Figure 5H). These findings suggest that semaglutide effectively promoted satiety and body weight reduction in *Bbs5^–/–^* mice.

**Figure 6:**
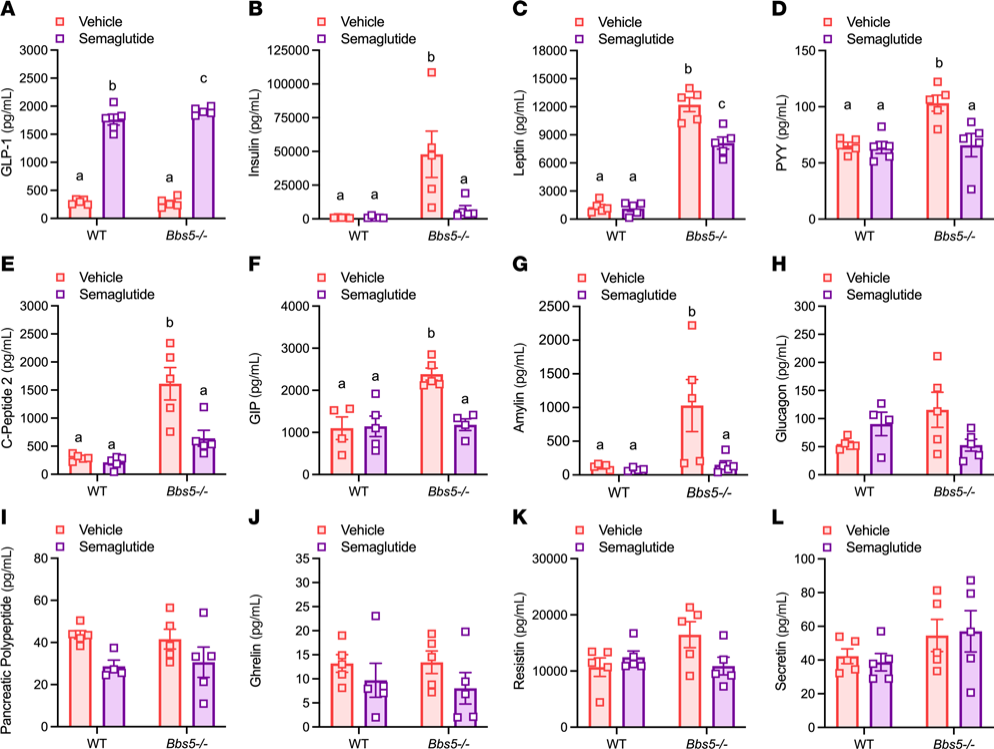
Semaglutide treatment improves dysregulated plasma levels of circulating metabolic hormones in adult *Bbs5*-null mice. (**A**–**L**) Plasma concentrations of GLP-1 (active) (**A**), insulin (**B**), leptin (**C**), PYY (**D**), C-peptide 2 (**E**), GIP (total) (**F**), amylin (**G**), glucagon (**H**), PP (**I**), ghrelin (**J**), resistin (**K**), and secretin (**L**) in 22- to 28-week-old female ad libitum chow-fed WT C57BL/6J and *Bbs5^–/–^* mice (*n* = 5 per group) before and after semaglutide therapy. Data in **A**–**L** were analyzed using 2-way ANOVA with Benjamini, Krieger, and Yekutieli post hoc test (FDR = 0.05) to compare individual time points. Data are mean ± SEM from WT and *Bbs5^–/–^* mice; means with no letters in common represent significantly different values (*P* < 0.05).

Beyond its metabolic benefits, semaglutide treatment significantly improved nest-building performance in *Bbs5^–/–^* mice (Figure 5I). This improvement was accompanied by enhanced glucose clearance, as demonstrated by a lower area under the curve during i.p. glucose tolerance tests (IPGTTs) (Figure 5, J and K). Similar benefits were observed in chow-fed WT mice; however, their nest-building performance was already at the maximal score, leaving no room for further improvement with semaglutide treatment (Supplemental Figure 6). Treatment with semaglutide significantly elevated plasma GLP-1 levels in both *Bbs5^–/–^* and WT mice (Figure 6A), confirming that intervention was effective regardless of genotype. In *Bbs5^–/–^* mice, semaglutide administration led to significant reductions in circulating plasma levels of insulin, leptin, PYY, C-peptide 2, GIP, and amylin (Figure 6, B–G) that in many cases normalized hormonal levels to those in WT littermates. No differences were observed in plasma levels of glucagon, PP, ghrelin, resistin, or secretin in *Bbs5^–/–^* mice before or after semaglutide treatment (Figure 6, H–L). Notably, semaglutide treatment did not affect the plasma concentrations of these metabolic hormones in healthy WT mice (Figure 6, B–L). These findings suggest GLP-1RAs hold promise for treating BBS and warrant further clinical investigation.

## Discussion

This study suggests that *Bbs5^–/–^* mice are a valuable model for BBS, recapitulating cardinal metabolic and neurobehavioral symptoms observed in patients. We demonstrate that *Bbs5* mutation disrupted energy homeostasis through impaired central nervous system processing of peripheral satiety cues potentially involving disruptions in adipose tissue and pancreatic function. Elevated levels of key hormones observed in untreated *Bbs5^–/–^* mice reflected significant metabolic disruptions consistent with insulin resistance, hyperleptinemia, and altered satiety signaling. These findings align with phenotypes commonly associated with BBS, including obesity and metabolic dysregulation.

By integrating behavioral phenotyping with hypothalamic transcriptomics, we identified dysregulation of leptin and CCK signaling pathways, which are central to energy homeostasis. Supporting the relevance of our model, a recent study using stem cell–derived hypothalamic arcuate-like neurons with induced BBS mutations has similarly shown impairments in leptin and insulin signaling (29), underscoring the role of primary cilia in energy homeostasis. Importantly, the gene expression changes observed in Bbs5^–/–^ mice are not replicated in mice with prolonged high-fat diet exposure or obesity (30), suggesting that the effects are due to *Bbs5* knockout rather than a secondary response to the metabolic changes. Impaired leptin receptor signaling is a primary mechanism driving weight gain (24, 26), and the role of CCK-A receptor signaling in satiety is well established (31). Evidence also supports a broader role for CCK-B receptors in modulating feeding behaviors, particularly in stress- or anxiety-related contexts. Prior studies demonstrated that selective antagonism of brain CCK-B receptors increases food intake and delays satiety, suggesting a role for CCK-B receptors in postprandial feeding regulation (32). Furthermore, elevated hypothalamic NPY levels in CCK-B receptor–knockout mice have been linked to hyperphagia, increased fat deposition, and obesity (33, 34). Our data showing blunted responses to exogenous CCK and downregulated hypothalamic *Cckbr* receptor in *Bbs5^–/–^* mice support a model in which impaired CCK signaling contributes to hyperphagia. In pre-obese *Bbs5^–/–^* mice, significant downregulation of *Lepr* and *Cck* expression suggests that disruptions in leptin and CCK signaling pathways precede weight gain. Hyperphagia in BBS appears to result from multifactorial disruptions, whereby intrinsic defects in CCK signaling pathways and receptor function further exaggerated obesity-related resistance to satiety signals (31). This concept is supported by our findings in obese *Bbs5^–/–^* mice, which exhibit additional downregulation of *Cck*, *Cckbr*, *Stat3*, and *Socs3* expression, indicating that impaired leptin and CCK signaling contributes to the hyperphagia observed in BBS. Together, these studies suggest that BBS disrupts CNS processing of satiety cues, contributing to hyperphagia and obesity.

While there are some similarities between diet-induced and genetic models of obesity, they differ significantly in insulin sensitivity and hormonal profiles, highlighting that obesity-induced effects are not equivalent to those driven by genetic predisposition (35). One limitation of our study is the absence of a lean/pair-fed *Bbs5^–/–^* group, which could have clarified whether the observed effects in ciliopathies are secondary to obesity. However, our findings are consistent with previous studies of other BBSome genetic knockouts (*Bbs2^–/–^* and *Bbs4^–/–^*), which demonstrate that dysregulated leptin receptor signaling is a key ciliopathy-mediated disruption contributing to obesity in BBS. Importantly, weight-matching through calorie restriction or pair-feeding to lean control body weights does not restore leptin signaling in these other BBS models, resulting in persistent hyperleptinemia and increased fat mass (24, 26). Compared with diet-induced obese models (23, 36, 37), *Bbs5^–/–^* mice exhibited distinct metabolic and immune characteristics, including rapid weight gain, pronounced immune dysregulation, and accelerated deterioration of glucose homeostasis and pancreatic function. While the models share hormonal changes and satiety impairments, *Bbs5^–/–^* mice displayed a unique immune profile characterized by elevated macrophages, a skewed M1/M2 balance, and Tregs with Th17-like phenotypes, resulting in a proinflammatory signature. These findings underscore disrupted adipose tissue homeostasis in *Bbs5^–/–^* mice, distinguishing them from diet-induced obesity models and emphasizing the importance of ciliopathy-specific mechanisms in metabolic dysfunction. Furthermore, primary cilia have been reported to cause changes in glucose homeostasis and insulin secretion in islet cells independently of body weight changes (16, 38, 39). This supports the hypothesis that ciliopathies, such as BBS, involve intrinsic defects in leptin and insulin pathways that are exacerbated, rather than solely caused, by obesity. However, further studies are warranted to determine whether metabolic dysfunctions, such as insulin resistance, are direct consequences of ciliopathy or secondary effects of obesity.

Our findings establish that GLP-1R signaling remained functional in *Bbs5^–/–^* mice, offering a compelling therapeutic target for BBS. This directly challenges the hypothesis by Shoemaker et al. (40) that GLP-1RAs may be ineffective in reducing appetite and body weight in the BBS population. In our study, treatment with semaglutide, a long-acting GLP-1RA, effectively mitigated core symptoms of BBS, including reduced food intake and decreased body weight, and improved glucose tolerance and neurobehavioral function. In *Bbs5^–/–^* mice, semaglutide normalized endocrine function, which may account for the observed improvements in insulin and leptin sensitivity, reduction in proinflammatory markers, and restoration of glucose tolerance. Collectively, these findings support the therapeutic potential of GLP-1RAs for managing BBS. This is further bolstered by a recent case report documenting significant weight loss in a BBS patient treated with a GLP-1RA (41).

Semaglutide exhibits broad metabolic and cognitive benefits that extend beyond weight loss. Prior studies show that semaglutide outperforms calorie restriction in diet-induced obese and pair-fed models, notably reducing pancreatic islet hypertrophy and enhancing β cell function (42). Pair feeding studies reveal that while calorie restriction promotes weight loss, it fails to reverse the broader spectrum of metabolic dysfunctions linked to obesity. By contrast, semaglutide has been shown to improve cognitive function, including enhancement of memory and learning, in both obese and nonobese rodent models (43). We hypothesize that semaglutide’s cognitive benefits are at least partially independent of weight loss, although concurrent metabolic improvements make it difficult to disentangle direct effects from secondary ones. Supporting this notion, Marinho et al. demonstrated that semaglutide produced weight-independent metabolic benefits in pair-fed diet-induced obese models (42). These findings underscore the potential of semaglutide to be an effective treatment option, but given the broad effects, there remains a need to establish optimal dosing regimens to ensure long-term safety and efficacy for pediatric and adult patients.

Our hypothalamic transcriptomics data revealed upregulated receptors, including calcitonin receptor, NPY2 receptor, and MC4R, providing insights into the neural mechanisms underlying metabolic dysregulation in *Bbs5^–/–^* mice. Downregulation of hypothalamic *Foxg1* and *Foxo3* expression may partially explain the impaired insulin signaling and glucose homeostasis observed in obese *Bbs5^–/–^* mice (44). Notably, the MC4R agonist setmelanotide (IMCIVREE) was recently approved by the US FDA to manage BBS-associated weight gain and is under evaluation for additional therapeutic effects in BBS patients (45, 46). However, hypersensitivity or allergic reactions to current medications, as well as defects in certain BBSome genes that disrupt NPY2 receptor trafficking to primary cilia, may limit the effectiveness of these therapies in specific BBS cases (47). Nevertheless, other receptor targets, such as the calcitonin receptor, present additional avenues for future targets in BBS that could serve as alternative effective therapeutics. Understanding these mechanisms could inform targeting of other aspects of the pathophysiology of BBS and similar ciliopathies, such as Alström syndrome.

Notably, the *Bbs5^–/–^* (*Bbs5^tm1b^*) mutation in our mice did not result in the increased mortality observed in *Bbs5^–/–^* (*Bbs5^tm1a^*) (48), *Bbs3^–/–^* (49), or *Bbip10^–/–^* mice (47), suggesting that the *Bbs5* mutation is not inherently lethal. This is consistent with the fact that there are patients with *BBS5* mutations who survive into adulthood. However, a previous study using the same *Bbs5^–/–^* (*Bbs5^tm1b^*) model reported abnormal retinal function (7) consistent with other BBS rodent models (50). *Bbs5^–/–^* mice exhibit significant visual impairments due to retinal degeneration, including a complete loss of cone photoreceptor function and reduced rod function, as evidenced by structural abnormalities in the outer nuclear layer and mislocalization of photoreceptor proteins. Importantly, these effects are age dependent, with retinal degeneration becoming pronounced only after 10 months of age (51). To avoid confounding our results with vision-related impairments, all experiments in this study were performed in younger mice, prior to the onset of severe retinal degeneration. Additionally, data from the MRC Harwell Institute indicate that sex differences may exist, with female mice showing fewer or less-severe visual defects compared with males. These findings emphasize the need to account for age and sex differences in experimental designs, and they highlight the limitations of the *Bbs5*-knockout model in fully replicating the visual phenotypes observed in human BBS. Our study illustrates the importance of studying ciliopathies, not only as rare genetic disorders but also as critical contributors to metabolic dysregulation in more prevalent forms of obesity. This is supported by recent evidence demonstrating that metabolic disorders such as type 2 diabetes (15, 52) and diet-induced obesity (30) are associated with downregulation in cilia genes — known to be involved in proliferation, cell cycle control, and cilia motility (1) — representing a potentially underexplored mechanism contributing to common obesity.

In conclusion, this work offers insights into the interplay between *Bbs5* deficiency, humoral and immune signaling, and metabolic dysregulation. The data support the development of GLP-1RAs as a promising therapeutic approach for managing the multifaceted clinical manifestations of BBS.

## Methods

### Sex as a biological variable

Both male and female mice were examined. Differences between sexes were evaluated to study mechanisms.

### Animals

WT C57BL/6J male mice (The Jackson Laboratory) and heterozygous *Bbs5^+/–^* mice that harbor a β-galactosidase (lacZ)–tagged, knockout allele of *Bbs5* in exon 4 and 5 (*Bbs5^tm1b(EUCOMM)Wtsi^*) maintained on a C57BL/6J background (MRC Harwell Institute) were used to produce *Bbs5^–/–^* mice. Upon arrival, mice were acclimated to housing at 22°C–24°C under a 12-hour light/12-hour dark cycle with ad libitum access to irradiated water and a low-fat chow diet (LFD; 3.1 kcal/g; Teklad 2018, Envigo) under pathogen-free conditions in the Animal Research Facility at the University of Florida. While both male and female heterozygous mice were fertile, no litters were produced when the homozygous *Bbs5^–/–^* mice were mated.

### Food intake, body weight, and body composition measurements

Food consumption was monitored manually using a weighing scale at the end of the dark period (more-active phase) and light period (less-active phase). Body weight was recorded between 10 am and noon using a conventional weighing scale, and body composition was measured in the unanesthetized mouse by a quantitative magnetic resonance method using an EchoMRI 700 Analyzer.

### Nesting behavior test

For nesting trials, each mouse was placed in the home cage with clean corncob bedding (approximately 150 g dry weight) and a single pressed cotton square at the end of the light cycle. Mice were allowed to form the nest, and photographs of nests formed after 12 hours and 24 hours were recorded for scoring analyses later. For analyses, at least 2 individuals masked to the study were provided the scoring criteria (adapted from ref. 53) and trained with baseline and example nests.

### NOR test

The NOR test was used to assess learning and memory in mice. The task was performed in the same chamber as the open field test. The NOR protocol consisted of 3 phases: habituation, familiarization, and test sessions. During the habituation session, mice were allowed to freely explore the empty open field for 5 minutes; 24 hours later, each mouse was returned to the arena, which contained 2 identical objects placed at symmetrical positions 5 cm from the arena wall and allowed to explore them freely for 10 minutes. During the familiarization session, most mice reached a minimum exploration total for both objects of 30 seconds. After a retention interval of 24 hours, the mouse was returned to the arena, where one of the objects was replaced by a novel object, and allowed to explore both the familiar object and the novel object for 10 minutes. All animal behavior tests were conducted in a room illuminated with standard fluorescent lights and digitally recorded with an overhead digital camera (HD Pro Webcam C920, Logitech). Analysis was performed using Noldus EthoVision XT videotracking software. Percent exploration time was calculated by dividing the time spent with either a familiar or a novel object by the total exploration time in the arena.

### Open field test

For 1 week before testing started, the mice were handled once a day for at least 5 minutes to reduce stress. The open field apparatus used in this study was a square arena (41 × 41 cm) with walls (height: 30.5 cm) to prevent the animals from escaping. Two regions are defined in the arena: the center, which accounts for 25% of the total area, and the periphery, which accounts for the remaining 75%. Tests were conducted during the dark phase, between 10 am and noon. Mice were placed in the center of the open field with lights on and then allowed to explore for 5 minutes. All animal behavior tests were conducted in a room illuminated with standard fluorescent lights and digitally recorded with overhead Logitech HD Pro Webcam C920 digital cameras. Analysis was performed using Noldus EthoVision XT videotracking software.

### EPM test

EPM testing was adapted from reported methods (54). The plus-shaped apparatus was made of painted wood and consisted of 2 opposite open arms and 2 opposite closed arms (30 × 5 cm) connected by a central platform (5 × 5 cm), with the arms and platform elevated 60 cm from the floor. To start the test, a naive mouse was placed at the center of the platform along the axis of the open arms, and its movements on the maze were recorded for 5 minutes with the overhead Logitech digital camera. The maze surfaces were then cleaned with 70% ethanol solution before placement of the next test mouse. Both the EPM and Y-maze tests were conducted in a room illuminated with standard fluorescence lights.

### Y- maze test

To begin the test, a naive mouse was placed at one corner of the light side with its head facing away from the opening. The mouse’s movements were digitally recorded for 8 minutes with an overhead digital camera (HD Pro Webcam C920, Logitech). The maze arm surface was cleaned with 70% ethanol solution before placement of the next test mouse.

For each behavioral test, mice in both groups were tested in a randomized order to offset potential test sequence bias. Test parameters were independently scored by 2–3 researchers masked to the experimental design. Mice were individually brought from the colony room to the behavioral room for the start of each behavioral test.

### IPGTT and insulin tolerance test

For IPGTT, after overnight fasting (~16 hours), an i.p. injection of 50% glucose (Sigma-Aldrich) solution at a dose of 2 g/kg body weight was administered to mice. For insulin tolerance tests, after a short fast (~6 hours), an i.p. injection of insulin (Humalog, Eli Lilly) at 0.5 IU/kg body weight was administered to mice. Blood glucose concentrations were determined from the tail vein using a hand-held glucometer (OneTouch UltraMini glucose meter; LifeScan) at 0, 15, 30, 60, and 120 minutes after glucose or insulin injections.

### Immunostaining of pancreas

Pancreases were dissected from 6 WT and 5 *Bbs5^–/–^* ad libitum fed male mice to assess the number of insulin-producing β cells. For tissue collection, following cardiac perfusion with PBS and 4% paraformaldehyde (PFA), the pancreas was removed and fixed in PFA for an additional 16 hours at 4°C. Following fixation, the tissue was washed with PBS and then embedded in paraffin. Pancreas sections (4 μm) were cut at 4 levels separated by 150 μm each (0, 150, 300, and 450 μm), with 2 serial sections placed per Superfrost Plus slide (ThermoScientific) at each level. The slides were stained using multiplex IHC (mIHC) according to methods similar to the mIHC described for human islets (55). All steps were performed at room temperature, except where noted. All primary antibodies were diluted in antibody diluent (Thermo Fisher Scientific), except for Ki-67, as described below. Sections were heated at 60°C for 1 hour, dewaxed in xylenes, and rehydrated in descending ethanols. Sections were subjected to antigen retrieval using preheated citrate buffer (BioGenex) for 20 minutes in a steamer, followed by cooling for 20 minutes. Endogenous peroxidase and alkaline phosphatase (AP) activity was blocked with 3% hydrogen peroxide for 10 minutes. Sections on the left side were blocked with mouse-on-mouse (M.O.M.) Ig blocking reagent (Vector, PK-2200) made up in diluent for 1 hour, followed by 5 minutes in M.O.M. diluent. The rabbit anti–Ki-67 antibody (Novus Bio, NB500-170) was diluted in M.O.M. diluent at 1:200 and incubated for 1 hour. To detect Ki-67 binding, a biotinylated goat anti-rabbit secondary antibody was used at 1:250 dilution for 30 minutes, followed by the ABC reagent (Vector, AK-5000) for 5 minutes. Sections on the right side were incubated with Sniper (Biocare, BS966M) for 10 minutes and rabbit anti-somatostatin (anti-SST) (Agilent/Dako, A0566) diluted at 1:1,000 for 1 hour. To detect SST binding, sections were incubated with goat anti-rabbit HRP conjugate (Biocare, RHRP520). The chromogen DAB was used to detect Ki-67 and SST for 1–6 minutes. Slides were then subjected to a second citraconic anhydride (Citra) antigen retrieval for 5 minutes, followed by 15 minutes cooling. Sections were then blocked with dual endogenous enzyme-blocking reagent (DEEB, Agilent/Dako, S200389-2) (10 minutes) and Avidin/Biotin/Sniper (Vector, SP-2001) (3 minutes each) in turn, followed by mouse anti-glucagon (Abcam, ab10988) (1:1,000, 30 minutes) and goat anti-mouse AP conjugate (Biocare Medical, MALPH521H) (30 minutes), and detected using Ferangi Blue (Biocare Medical, FB813H) (2 minutes). Sections were subjected to a third Citra antigen retrieval, followed by guinea pig anti-insulin (Agilent/Dako, A0564) (1:1,000, 30 minutes), biotinylated goat anti-guinea pig IgG (Vector, BA-7000) (30 minutes), and Warp Red chromogen (Biocare Medical, WR806) (1 minute). Sections were counterstained with hematoxylin (Biocare Medical) at 1:10 for 30 seconds, followed by air drying and mounting.

### Blood collection and hormone analysis

Blood samples were collected 2–3 hours after dark onset from the tail vein of mice that had ad libitum food access. The blood samples were collected in tubes containing EDTA (1.5 mg/mL blood), protease inhibitor cocktail (10 mL/mL blood; Sigma-Aldrich), and dipeptidyl peptidase IV inhibitor (DPP-IV inhibitor, 10 mL/mL blood; Millipore) and centrifuged for separation of plasma, which was stored at –80°C until analysis.

Plasma concentrations of amylin (active), C-peptide 2, ghrelin, GIP (total), GLP-1 (active), glucagon, insulin, leptin, PP, PYY, resistin, and secretin were measured in duplicate by Eve Technologies using a commercially available Mouse Metabolic Hormone 12-Plex Discovery Assay (MilliporeSigma) according to the manufacturer’s protocol on a Luminex 200 system (Thermo Fisher Scientific). Assay sensitivities of these markers range from 1.4 to 91.8 pg/mL for the 12-plex. Individual analyte sensitivity values are available in the MilliporeSigma MILLIPLEX MAP protocol.

### Isolation of the stromal-vascular fraction from eWAT

The stromal vascular fraction was isolated from the visceral adipose tissue as previously described (Figure 3A) (56). Briefly, eWAT was harvested and weighed. eWAT explants (500 mg) were digested in 1 mL DMEM supplemented with 2% fatty acid–free BSA (Sigma-Aldrich, 126575), HEPES (10 mM), Liberase TM (thermolysin medium) (25 μg/mL) (Roche, 05401119001), and DNAse (250 μg/mL) (Roche, 10104159001) for 1 hour at 37°C. Digested tissue was filtered through a 150 μm mesh into preheated DMEM containing 2% FBS. Cells were spun down at 4°C, 500*g*, for 10 minutes. Cell pellets were suspended in 0.5 mL ammonium-chloride-potassium (ACK) buffer to lyse contaminating erythrocytes. Cells were spun and collected at 2,200*g* for 5 minutes at 4°C. Cells were resuspended in FACS buffer.

### Adipose tissue immunophenotyping and flow cytometry

Single-cell suspensions from eWAT samples from mice of both groups and both sexes were stained with fluorescent dye–conjugated antibodies in FACS buffer (PBS containing 2% FBS and 1 mM EDTA) (56). For intracellular cytokine or transcription factor staining, cells were fixed and permeabilized with the *Foxp3* staining buffer set (eBioscience, 00-5523-00). Data were acquired on a BD LSRFortessa and analyzed using the FlowJo software package. Cell sorting was performed on a BD FACSAria III flow cytometer and cell sorter. The following flow antibodies were used: anti-mouse CD4 PE/Cy7 (clone GK1.5) (BioLegend, 100422), anti-mouse IL-17a PE (clone TC11-1810.1) (BioLegend, 506903), anti-mouse CD45 PerCP/Cy5.5 (clone 30-F11) (BioLegend,103131), anti-mouse GATA3 (BioLegend, 653809), anti–mouse/human CD11b PE/Cy7 (clone M1/70) (BioLegend, 101216), anti-mouse/human CD11b Brilliant Violet 605 (clone M1/70) (BioLegend, 101237), anti–mouse inducible NO synthase (iNOS) (Invitrogen, 125920), anti–mouse Foxp3 Pacific blue (clone MF-14) (BioLegend, 26410), anti–mouse/human Arg1 FITC (R&D Systems, IC5868F), anti-mouse F4/80 PerCP/Cy5.5 (clone BM8) (BioLegend, 123127), anti–mouse MGL2/CD301B (BioLegend, 146807), anti–mouse/human T-bet PE/Cy7 (clone 4B10) (Biolegend, 644824), anti–mouse/human RORγt (APC, clone AFKJS-9) (Invitrogen, 17698882), anti–mouse Ly6G (clone 1A8) (BioLegend, 127613), and PE anti–mouse CD206 (clone C068C2) (BioLegend, 141705).

### Core hypothalamic tissue dissection

For the RNA-Seq experiment, core hypothalamic tissues of 5-week-old (pre-obesity) and 12-week-old (3 *Bbs5^–/–^* and 2 WT littermates per age group) mice were collected using a brain tissue slicer and micropuncher (Integra-Miltex). Briefly, the head was decapitated by cutting posterior to the ears, and a midline skin incision was made caudal to the sagittal suture, with care taken not to cut through the brain. A small cut was made through the anterior of the skull between the eyes. Parietal bones were tilted to expose the brain with meninges. The brain was freed from the meninges and gently lifted from the skull by curved narrow forceps. It was then placed on a precooled petri dish and transferred onto ice. The brain was placed on a precooled brain slicer and sliced using 2 blades. The tissue slice was removed and transferred to a Sylgard-coated petri dish with ice-cold sterile PBS. Part of the hypothalamus was isolated using an Integra Miltex disposable biopsy punch. The punched tissue was immediately transferred onto a labeled Eppendorf tube and snap-frozen on dry ice.

### Hypothalamic RNA-Seq

The libraries for RNA-Seq were prepared with a KAPA Stranded RNA-Seq Kit (Roche). Integrity and quantification of RNA were assessed using a 4200 TapeStation Instrument (Agilent Technologies). 250 ng RNA was taken for RNA-Seq library preparation. Sample libraries were prepared with the NEBNext Ultra Directional RNA Library Prep for Illumina kit (New England Biolabs). The workflow consisted of mRNA enrichment, cDNA generation, and end repair to generate blunt ends, A-tailing, adaptor ligation, and PCR amplification. Sequencing was performed on an Illumina Hiseq3000 for a single-read 50 runs.

#### Data processing.

The quality of FASTQ files was checked using fastQC, and then the reads were aligned to GRCh38 bt TopHat and Bowtie (57). The BAM files outputted by TopHat were quality checked by RSeQC (58) and mapped by SAMtools (https://www.htslib.org/). The reads of the filtered BAM files were counted using featureCounts (59). Processing of RNA-Seq data was performed by Adrien Jeanniard (UCLA Scientific Core Services, Los Angeles, California USA). RNA-Seq analyses were done by use of DESeq2 (60), a method for differential analysis of count data using shrinkage estimation for dispersions and fold changes (FCs) to improve stability and interpretability of estimates. In brief, normalized counts were calculated by dividing raw read counts by sized factors and fitted to a negative binomial distribution, followed by the generalized linear model (GLM) likelihood ratio test (61). Statistical significance (*P* values) for differentially expressed genes (DEGs) were first corrected by using the R fdrtool (v.1.2.15) package and then adjusted for multiple testing with the Benjamini-Hochberg correction. 9,300 transcripts were removed, as the samples lacked those expressions. Differential expression ratio and log_2_ FC were calculated for each significant gene. Downregulation was indicated by negative FC, whereas positive values represented upregulations.

### Acute drug studies

#### Leptin.

Animals were fasted overnight (~12–14 hours) before receiving an i.p. injection 1 hour in the dark period of either 0.1% wt/vol BSA (vehicle) or recombinant murine leptin (2 mg/kg body weight; R&D Systems) and monitored for changes in food intake and body weight over 24 hours.

#### CCK-8.

Animals were fasted overnight (~12–14 hours) before receiving an i.p. injection 1 hour in the dark period of either 0.1% wt/vol BSA (vehicle) or CCK-8 sulfated (2 or 4 μg/kg body weight; Tocris Bioscience) and monitored for changes in food intake and body weight over 24 hours.

#### Exendin-4.

Animals were fasted overnight (~12–14 hours) before receiving an i.p. injection 1 hour in the dark period of either 0.1% wt/vol BSA (vehicle) or the GLP-1RA exendin-4 (0.1, 1, or 2 μg/kg body weight; Tocris Bioscience). They were then monitored for changes in food intake and body weight over 24 hours.

### Semaglutide study

To determine the therapeutic potential of GLP-1RA on body composition, food intake, and glucose tolerance, we subcutaneously injected mature animals (18–30 weeks old) with either vehicle (6% vol/vol DMSO in 0.9% saline) or semaglutide (0.15 mg/kg body weight; gift from Novo Nordisk) in a crossover study design (Figure 5A). Mice were injected with the drugs once daily at the onset of dark period and fed ad libitum for 2 weeks. Animals were monitored daily for changes food intake and body weight and weekly for changes in body composition, and underwent biweekly glucose tolerance tests.

### Statistics

Statistical analysis for the experiments is described in the figure legends and was performed using GraphPad Prism 8.3 software. One-way ANOVA, with or without repeated measures, was used for comparing groups; 2-way ANOVA, with or without repeated measures, was used for comparing more than one factor between groups, as performed for food intake, fat mass, lean mass, body weight, weight gain, and blood glucose during IPGTT and insulin tolerance tests. Mixed-model analysis was used in case there were missing data. The sample size (*n*) for each experiment is shown in the figure legends and corresponds to the sample derived from the individual mice. Data are presented as mean ± SEM, and a *P* value less than 0.05 was considered statistically significant.

### Study approval

Experiments on animals were performed in accordance with the *Guide for the Care and Use of Laboratory Animals of the NIH* (National Academies Press, 2011). All mouse experiments were performed according to the regulations of and with approval by the Institutional Animal Care and Use Committee of the University of Florida (protocol 202110305).

### Data availability

All data points shown in graphs, as well as the RNA-Seq differential fold change dataset, are available in the Supporting Data Values file. All RNA transcriptomics data generated for this manuscript have been deposited in the NCBI’s Gene Expression Omnibus database (GEO GSE293590).

## Author contributions

AS, and GL conceived the study design. AS, GL, and SCS secured funding. AS, NH, MY, SL, SM, LJ, and MCT performed the experiments. AS, NH, MY, SM, GL, LJ, and SCS analyzed the data, and AS and GL wrote the manuscript. All authors reviewed and edited the manuscript and had final approval of the submitted version.

## Supplementary Material

Supplemental data

Supporting data values

## Version 1 04/15/2025

In-Press Preview

## Version 2 06/16/2025

Electronic publication
