# Supplementary material for: Transcriptome-guided GLP-1 receptor therapy rescues metabolic and behavioral disruptions in a Bardet-Biedl syndrome mouse model
Source: J Clin Invest. 2025 Apr 15;135(12):e184636. doi: 10.1172/JCI184636 (PMC12165790; doi:10.1172/JCI184636)
Supplement: Supplemental data [file jci-135-184636-s007.pdf]

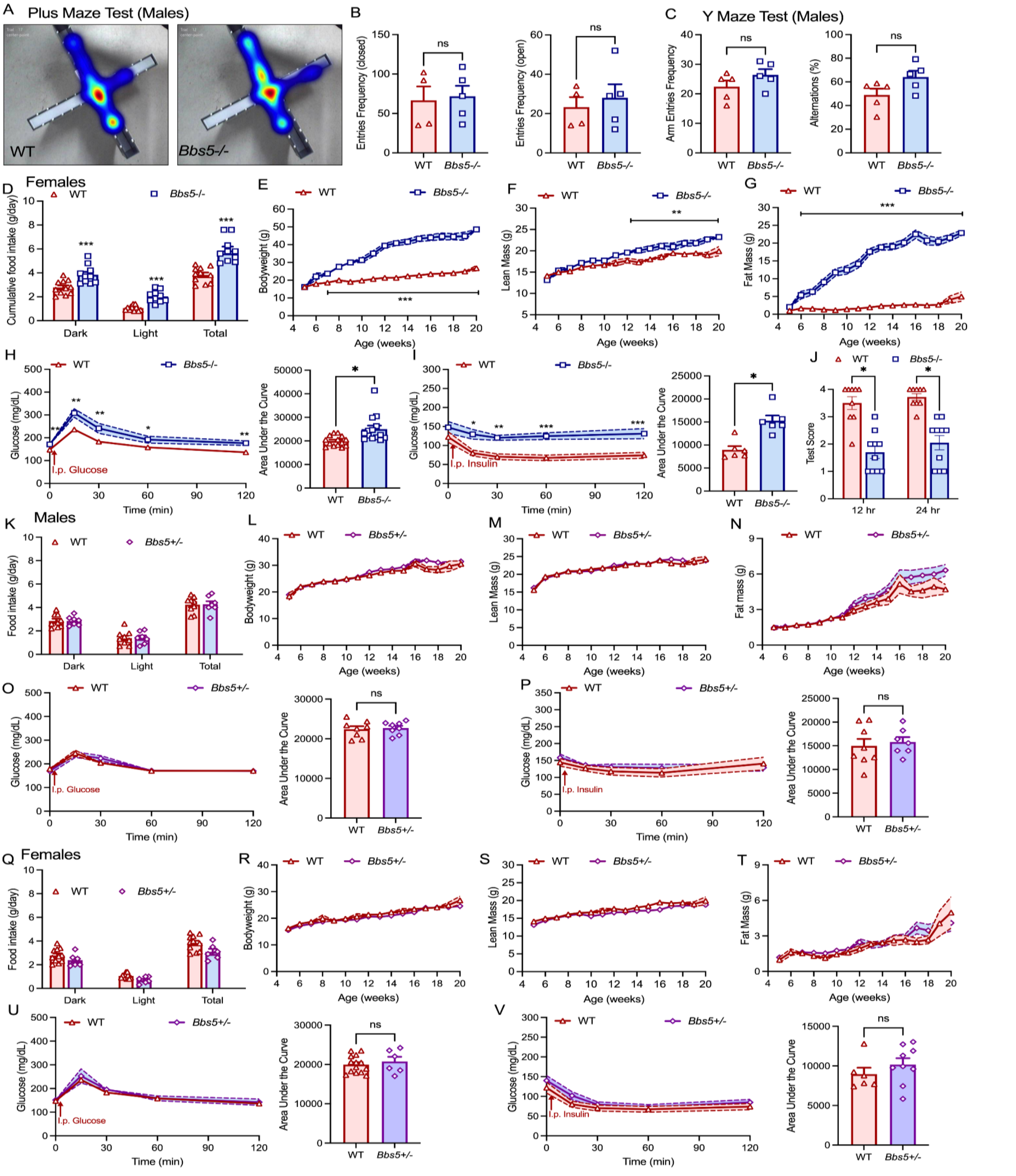

Supplementary Figure. 1| Female *Bbs5* null mice are morbidly obese, hyperphagic, glucose intolerant, and have cognitive impairments while male and female heterozygous *Bbs5 (+/-)* were no different compared to WT controls. A-B, Representative plus maze heatmaps from littermate wildtype (WT) and *Bbs5-/-* male mice respectively (**A**) and number of entries in closed and open arms (**B**). **C**, Y-maze arm entries frequency, and 5-min elevated in 10-18-weeks-old male mice (n= 5 per group). The data in **D-J** are from female mice. **D**, Average cumulative daily *ad libitum* food intake during 12-hour dark and light, and 24-hour periods in 10-18-weeks-old female mice (n= 10-11 per group). **E-G**, Weekly body weight (**E**), lean mass (**F**), and fat mass (**G**) development in chow-fed 5- 20-weeks-old female WT and *Bbs5-/-* mice (n= 5-11 per group). **H-I**, Intraperitoneal glucose tolerance and its area under the curve (**H**) and insulin tolerance and its area under the curve (**I**) in 11-18-week-old female WT and *Bbs5-/-* mice (n= 6-8 per group). **J,** Nest building score after 12 and 24 hours upon providing cotton-pressed nestlet in 12-19-weeks-old female mice (n= 9-10 per group). The data in **K-V** are from heterozygous *Bbs5+/-* and WT mice. **K**, Average cumulative daily *ad libitum* chow intake during 12-hour dark and light, and 24-hour periods in 10-18-weeks old male WT and *Bbs5+/-* mice (n= 10-11 per group, 9 to18 weeks old mice). **L-N**, body weight (**L**), lean mass (**M**), and fat mass (**N**) development in chow-fed 5 – 20-weeks-old WT and *Bbs5+/-* male mice (n= 5-12 per group). **O-P**, Intraperitoneal glucose tolerance and its area under the curve (**O**), and insulin tolerance and its area under the curve (**P**) in 11-18-week-old WT and *Bbs5+/-* male mice (n= 6-8 per group). **Q**, Average cumulative daily *ad libitum* chow intake during 12-hour dark and light, and 24-hour periods in 10-18-weeks old female WT and *Bbs5+/-* mice (n= 10-11 per group). **R-T**, body weight (**R**), lean mass (**S**), and fat mass (**T**) development in chow-fed 5 – 20-weeks-old WT and *Bbs5+/-* female mice (n= 5-12 per group). **U-V**, Intraperitoneal glucose tolerance and its area under the curve (**U**), and insulin tolerance and its area under the curve (**V**) in 11-18-week-old WT and *Bbs5+/-* female mice (n= 6-8 per group). Data in **B-C** were analyzed using Student's two-sided, two-tailed *t*-test. Data in **D-V** were analyzed using repeated measures two-way analysis of variance (ANOVA) with Benjamini, Krieger, and Yekutieli post hoc test (FDR = 0.05) to compare individual time points. Data are mean ± s.e.m.; ns, not significant; **P*<0.05; ***P*<0.01; ****P*<0.001.

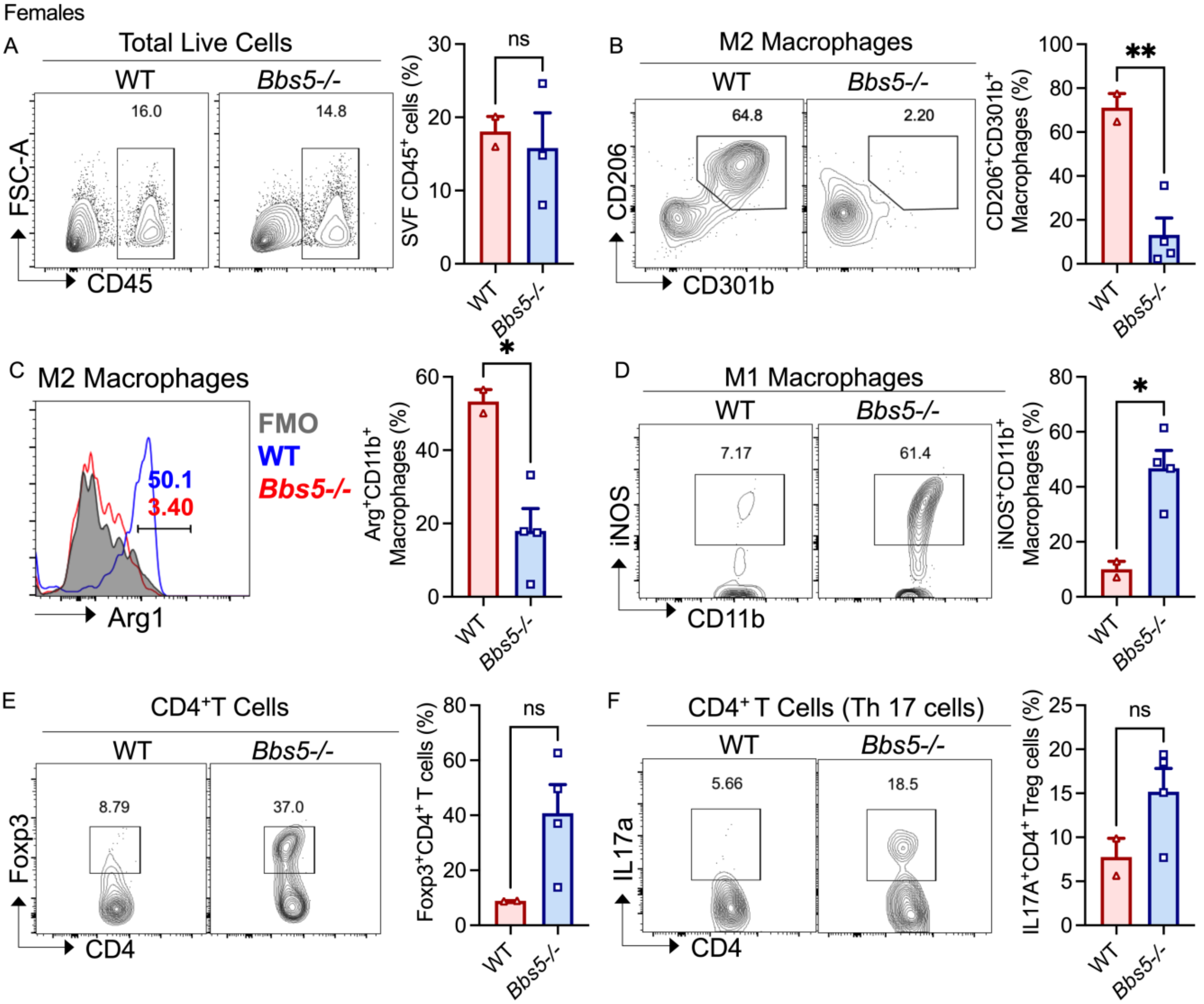

Supplementary Figure. S2| Female *Bbs5* null mice have proinflammatory white adipose immunophenotype. Flow cytometry analysis of CD45⁺ cells (**A**), CD206⁺CD301b⁺ M2 macrophages (**B**), Arg⁺ M2 macrophages (**C**), iNOS⁺ M1 macrophages (**D**), Foxp3⁺ Tregs (**E**), IL17⁺ Tregs (**F**) in eWAT of 18-21 weeks old WT and *Bbs5-/-* male mice (n= 2-4 per group). Data are representative of two independent experiments. Data in **A-F** were analyzed using Student's two-sided, two-tailed *t*-test. Data are mean ± s.e.m. from WT and *Bbs5-/-* female mice; ns, not significant; *P<0.05; **P<0.01.

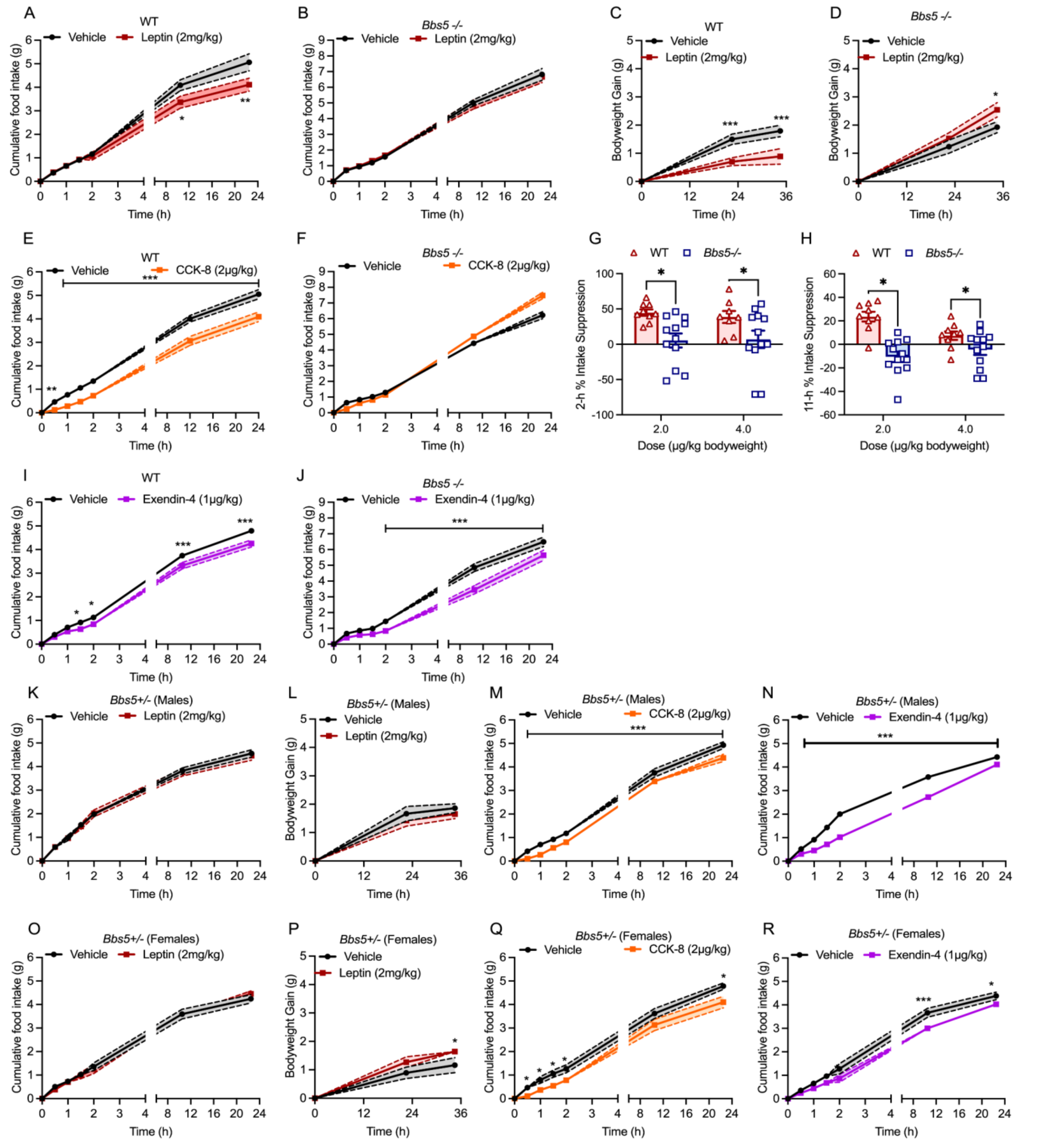

Supplementary Figure. S3| Adult *Bbs5* null female mice display leptin and cholecystokinin (CCK) resistance but retained glucagon-like peptide-1 (GLP-1) response while heterozygous *Bbs5* (+/-) male and female mice display leptin resistance but responded to CCK-8 and GLP-1R agonist. The data in **a-j** are from 12-18-weeks-old female WT and *Bbs5* null mice. **A-B**, the effect of intraperitoneal leptin (red, 2 mg/kg bodyweight) or vehicle (black) injection on cumulative *ad libitum* food intake in female WT (**A**) and *Bbs5-/-* mice (**B**) over 24 hours (n= 8-10 per group). **C-D**, body weight gain in WT (**C**) and *Bbs5-/-* (**D**) at 24- and 36-hour after leptin injections. **E-F**, the effect of intraperitoneal cholecystokinin (CCK-8, orange, 2 μg/kg bodyweight) or vehicle (black) injection on cumulative *ad libitum* food intake in female WT (**E**) and *Bbs5-/-* mice (**F**) over 24 hours (n= 9-12 per group). **G-H**, Food intake suppression relative to saline after 2-hour (**G**) or 11-hour (**H**) in female WT and *Bbs5-/-* mice following CCK-8 (2 or 4 μg/kg bodyweight) (n= 9-12 per group). **I-J**, the effect of intraperitoneal exendin-4 (GLP-1 agonist, purple, 1 μg/kg bodyweight) or vehicle (black) injection on cumulative *ad libitum* food intake in female WT (**I**) and *Bbs5-/-* mice (**J**) over 24 hours (n= 6-9 per group). The data in **K-N** are from 12-18-weeks-old male heterogenous *Bbs5+/-* mice (n=8 per group). **K**, the effect of intraperitoneal leptin (red, 2 mg/kg bodyweight) or vehicle (black) injection on cumulative *ad libitum* food intake in *Bbs5+/-* mice over 24 hours. **L**, body weight gain in *Bbs5+/-* at 24- and 36-hour after leptin injections. **M**, the effect of intraperitoneal cholecystokinin (CCK-8, orange, 2 μg/kg bodyweight) or vehicle (black) injection on cumulative *ad libitum* food intake in *Bbs5+/-* mice over 24 hours. **N**, the effect of intraperitoneal exendin-4 (GLP-1 agonist, purple, 1 μg/kg bodyweight) or vehicle (black) injection on cumulative *ad libitum* food intake over 24 hours in *Bbs5+/-* mice. The data in **O-R** are from 12-18-weeks-old female heterogenous *Bbs5+/-* mice. **O**, the effect of intraperitoneal leptin (red, 2 mg/kg bodyweight) or vehicle (black) injection on cumulative *ad libitum* food intake *Bbs5+/-* mice over 24 hours (n= 6 per group). **P**, body weight gain in *Bbs5+/-* at 24- and 36-hour after leptin injections. **Q**, the effect of intraperitoneal cholecystokinin (CCK-8, orange, 2 μg/kg bodyweight) or vehicle (black) injection on cumulative *ad libitum* food intake in *Bbs5+/-* mice over 24 hours (n= 6 per group). **R**, the effect of intraperitoneal exendin-4 (GLP-1 agonist, purple, 1 μg/kg bodyweight) or vehicle (black) injection on cumulative *ad libitum* food intake over 24 hours in *Bbs5+/-* mice (n= 5 per group). Data in **A-R** were analyzed using repeated measures two-way analysis of variance (ANOVA) with Benjamini, Krieger, and Yekutieli post hoc test (FDR = 0.05) to compare individual time points. Data are mean ± s.e.m. from vehicle or drug-treated mice; **P*<0.05; ***P*<0.01; ****P*<0.001

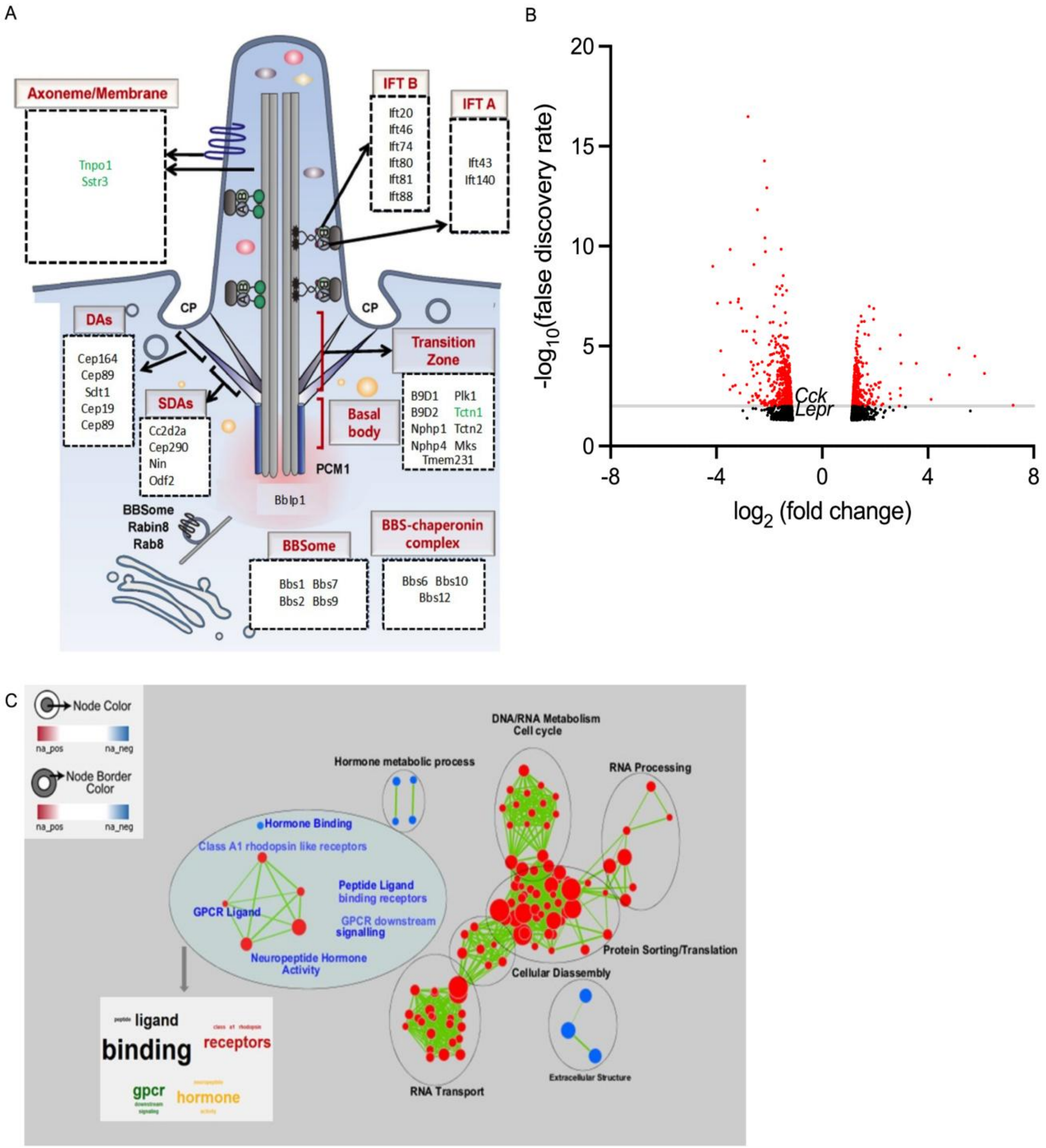

Supplementary Figure S4. Analysis of RNA-seq of the hypothalamus of pre-obese *Bbs5-/-* mice. A, Primary cilia genes dysregulated at 5 weeks as a result of *Bbs5* knockout. Upregulated genes are annotated in green. Dysregulated genes are shown with each component of primary cilia. Adapted from H. Lee et al (H. Lee et al., 2015). **B**, Volcano plot of differential expression changes, plotting the log fold change of receptors in the hypothalamus between 5-week-old WT and *Bbs5-/-* male mice (n=2-3 per group). Red dots are significant, black are non-significant. **C**, Enrichment map analyses RNASeq data shows 130 gene sets are enriched out of 924 gene sets. Protein sorting and translation, cellular disassembly, RNA transport, DNA and RNA metabolism and GPCR signalling pathway, neuropeptide receptor interactions, and ligand binding, are the upregulated gene sets (red). Downregulated gene sets include extracellular structure and hormone metabolic process (blue).

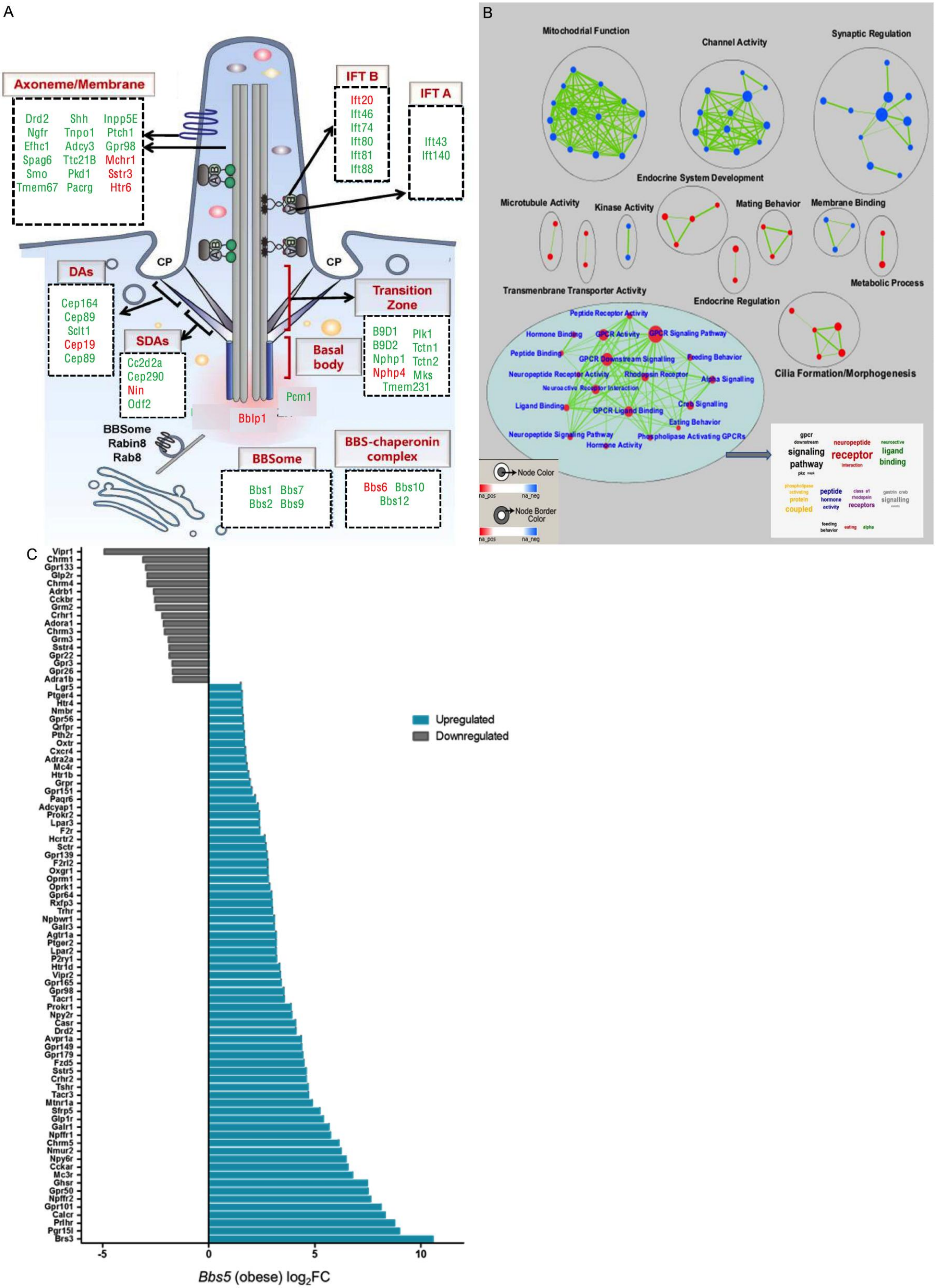

Supplementary Figure S5. Analysis of RNA-seq of the hypothalamus of obese *Bbs5-/-* mice. A, Primary cilia genes dysregulated at 12 weeks as a result of *Bbs5* knockout. Upregulated genes are annotated in green and downregulated gene in red. Dysregulated genes are shown in relation to each component of primary cilia. Adapted from H. Lee et al (H. Lee et al., 2015). **B**, Enrichment map analyses RNASeq data shows 111 gene sets are enriched out of 184 gene sets. GPCR signalling pathway, neuropeptide receptor interactions and ligand binding, cilia formation, metabolic process and endocrine system are the upregulated gene sets (red). Downregulated gene sets include synaptic regulation, channel activity, mitochondrial function and enzyme activity (blue). **C**, Plot showing 87 genes for GPCRs were detected to be differentially expressed (P<0.05, log2FC 1.5) in the hypothalamus between *Bbs5-/-* and WT mice.

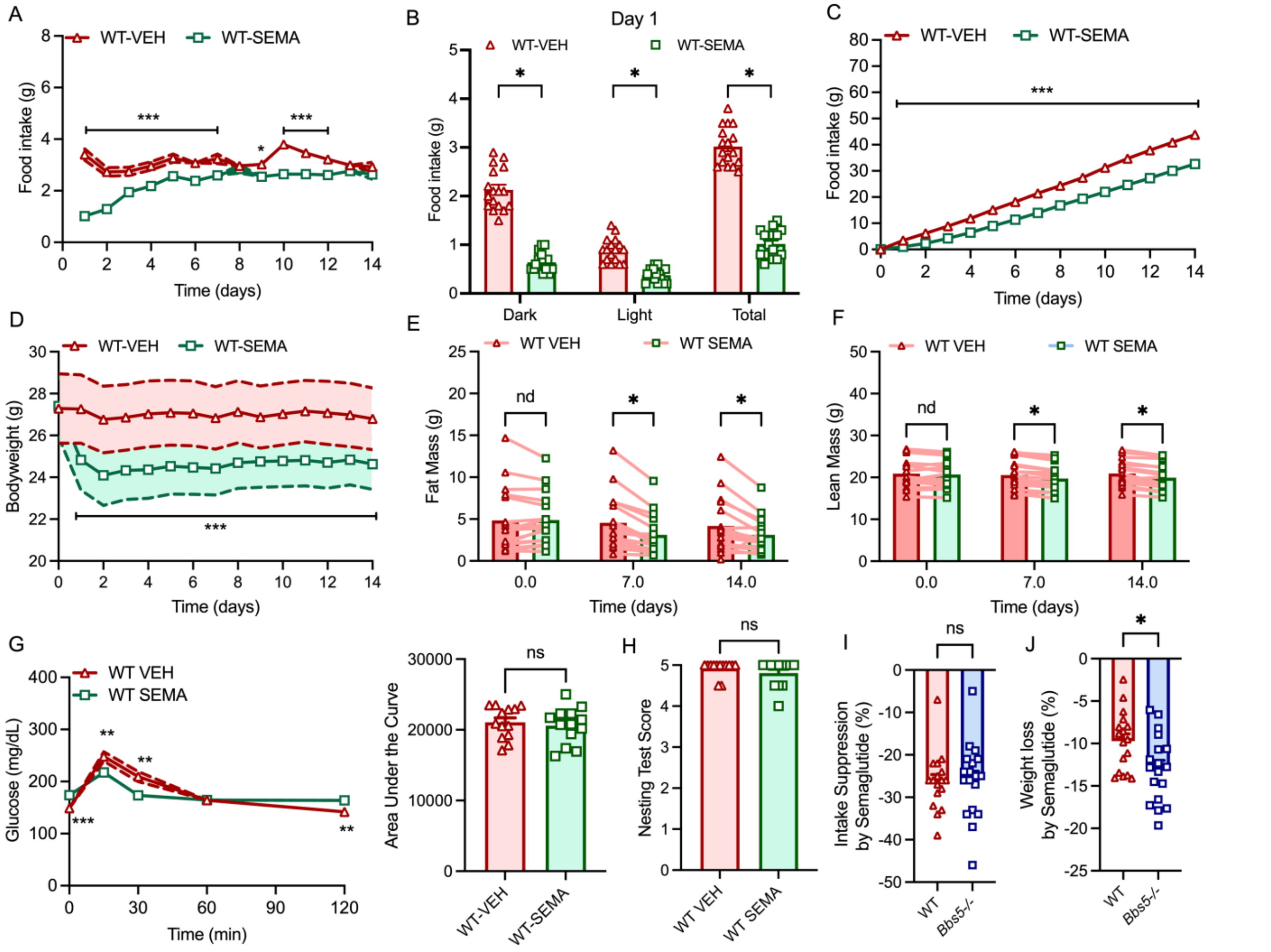

Supplementary Figure. S6| Glucagon-like peptide-1 agonist (Semaglutide) promotes hypophagia-induced weight loss and improves cognitive behavior and glucose tolerance in adult C57bl/6j wildtype mice. Adult *ad libitum* chow-fed WT mice received daily subcutaneous injections of vehicle (red) for 14 days followed by semaglutide (green, 0.15 mg/bodyweight) for 14 days and observed for changes in feeding, body composition, and glucose tolerance. The data in **A-H** are from 19-30-weeks-old WT mice (n=17 includes 6 males and 11 females). **A-C,** changes in *ad libitum* chow intake daily over 24 hours (**A**), average cumulative intake during 12-hour dark and light, and 24-hour periods (**B**), and average cumulative daily food intake during 14 days of vehicle or semaglutide therapy (**c**). **D-G,** average daily changes in body weight (**D**), weekly changes in fat mass (**E**), lean mass (**F**), and intraperitoneal glucose tolerance test (**G**) after semaglutide therapy. **H,** Nest building score after 24 hours upon providing cotton-pressed nestlet on vehicle and after semaglutide therapy. **I,** Average suppression in food intake and **J,** Average weight loss before and after semaglutide therapy in *Bbs5-/-* and WT mice. Data in **A-G** were analyzed using repeated measures two-way analysis of variance (ANOVA) with Benjamini, Krieger, and Yekutieli post hoc test (FDR = 0.05) to compare individual time points. Data in **H-J** were analyzed using Student's two-sided, two-tailed t-test. Data are mean ± s.e.m. from vehicle or drug-treated mice; *P<0.05; **P<0.01; ***P<0.001.
